# Supplementary material for: Therapeutic vaccination strategy targeting the PreS1 region of L-HBsAg for HBV/HDV co-infection
Source: JHEP Rep. 2026 Aug 17;8(11):101997. doi: 10.1016/j.jhepr.2026.101997 (PMC13659496; doi:10.1016/j.jhepr.2026.101997)
Supplement: Supplementary file 3 [file mmc4.pdf]

JHEP|Reports

Therapeutic vaccination strategy targeting the PreS1 region of L-HBsAg for HBV/HDV co-infection

Authors

Rebecca A. Russell, James Lok, James M. Harris, …, Sumi Biswas, Jane A. McKeating, Matthew D.J. Dicks

Correspondence

jane.mckeating@ndm.ox.ac.uk (J.A. McKeating), matthew.dicks@spybiotech.com (M.D.J. Dicks).

Graphical abstract

- A therapeutic vaccine strategy for chronic HBV infection was designed to target the PreS1 region of L-HBsAg, required for cell entry of HBV and HDV
- Adenovirus vectors encoding L-HBsAg and HBcAg were covalently decorated with PreS1-peptide using Tag/Catcher protein superglue

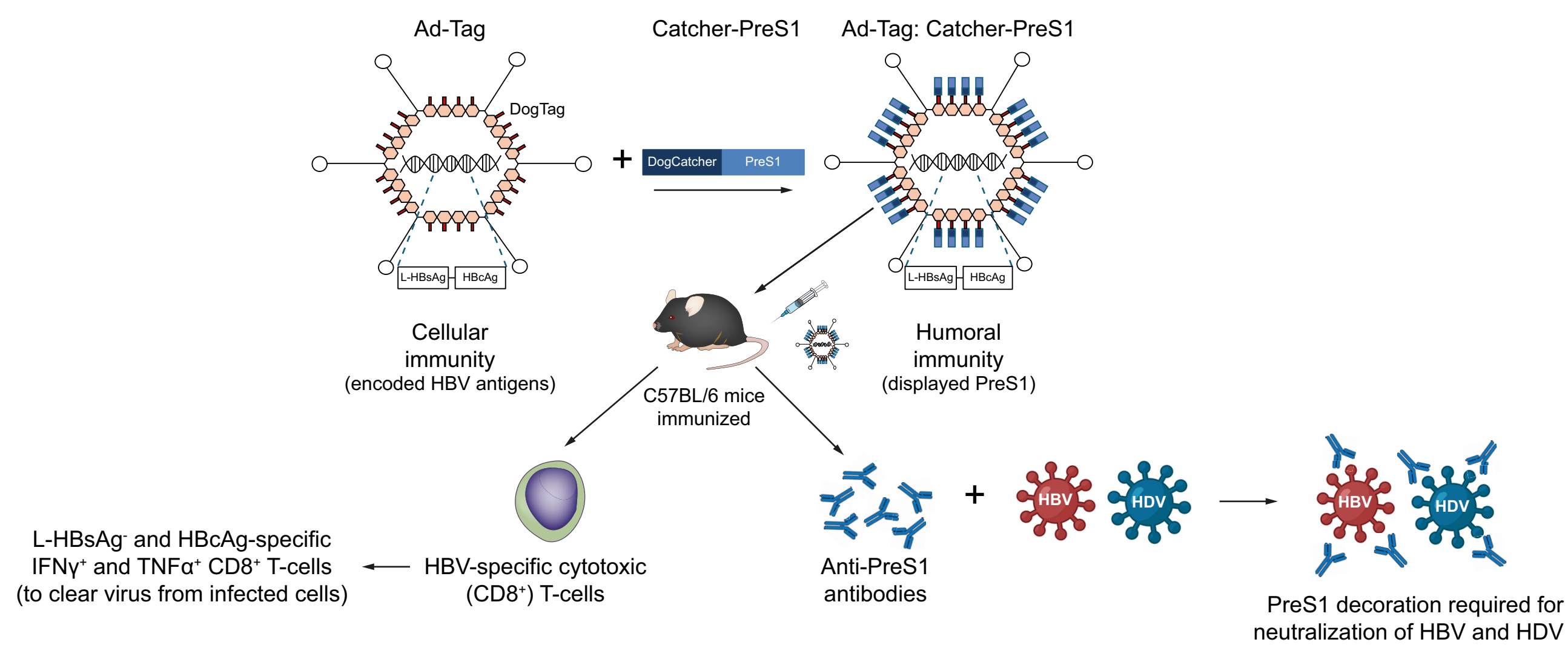

- **PreS1-decorated adenovirus combines HBV and HDV entry inhibition with anti-HBV T-cell induction in a single vaccine platform**

Highlights:

- PreS1 antibody responses are rare or absent in chronic HBV infection.
- PreS1-decorated adenovirus vectors induced serum neutralising antibody responses against HBV and HDV.
- PreS1-decorated adenovirus induced strong $CD8^+$ and $CD4^+$ T-cell responses against multiple encoded HBV antigens.
- PreS1-decorated adenovirus combines immunological entry inhibition with T-cell induction in a single vaccine platform.

Impact and implications:

A functional cure for chronic hepatitis B is urgently needed, and given the complexity of the disease, achieving this will likely require combination therapies that target multiple stages of the viral lifecycle. Here, we developed a therapeutic vaccination strategy that combines PreS1-targeted entry inhibition with induction of T-cell responses against multiple HBV antigens by decorating adenovirus particles encoding L-HBsAg and HBcAg with PreS1 peptide. In mice, PreS1-decorated adenovirus particles induced serum neutralising activity against both HBV and HDV and robust T-cell responses against encoded HBV antigens. Our findings suggest that PreS1-decorated adenoviral vaccines could complement existing therapeutic approaches for viral hepatitis.

https://doi.org/10.1016/j.jhepr.2026.101997

© 2026 The Authors. Published by Elsevier B.V. on behalf of European Association for the Study of the Liver (EASL). This is an open access article under the CC BY license (http://creativecommons.org/licenses/by/4.0/).

JHEP|Reports

Therapeutic vaccination strategy targeting the PreS1 region of L-HBsAg for HBV/HDV co-infection

Rebecca A. Russell[1], James Lok[2], James M. Harris[3], Senko Tsukuda[4], Louisa M. Rose[1], Bethany G. Charlton[1], Ivana Carey[2], Kosh Agarwal[2], Peter A.C. Wing[4], Sumi Biswas[1,5], Jane A. McKeating[3,4,*], Matthew D.J. Dicks[1,*]

JHEP Reports **2026**. vol. 8 | 1–12

Check for updates

Background & Aims: Achieving functional cure for chronic hepatitis B (CHB) will likely require a combinatorial approach targeting multiple aspects of the complex HBV life cycle and host adaptive immune responses. Here, we developed a therapeutic vaccination strategy targeting the PreS1 region of L-HBsAg, required for cellular entry of both HBV and HDV.

Methods: An established, potent T-cell-inducing platform, recombinant adenovirus (Ad), was used as a nanoparticle scaffold for PreS1 attachment to generate antibodies that neutralise virus entry and establish T-cell-mediated immune control. Ad particles encoding multiple HBV antigens were decorated with the PreS1 peptide using the DogTag/DogCatcher protein superglue. The immunogenicity of PreS1-decorated Ad was assessed in C57B/6 mice using IgG ELISA, serum neutralisation assays, INF-γ ELISpot, and intracellular cytokine staining.

Results: Screening a cohort of 61 patients diagnosed with CHB revealed minimal evidence of natural anti-PreS1 responses. Mice vaccinated with PreS1-decorated Ad induced robust anti-PreS1 antibody responses that neutralised HBV (p <0.01) and HDV (p <0.05) infections. By contrast, an undecorated Ad encoding L-HBsAg failed to neutralise HBV, demonstrating that PreS1 decoration was required for potent neutralisation of HBV. Strong $CD8^+$ and $CD4^+$ T-cell responses were induced against HBV antigens encoded in the Ad genome.

Conclusions: PreS1-decorated Ad combines immunological inhibition of HBV and HDV entry with potent anti-HBV T-cell induction in a single platform, providing a promising addition to current therapeutic strategies against CHB, with particular utility in HBV/HDV co-infection.

© 2026 The Authors. Published by Elsevier B.V. on behalf of European Association for the Study of the Liver (EASL). This is an open access article under the CC BY license (http://creativecommons.org/licenses/by/4.0/).

Introduction

In 2022, the WHO estimated that ~254 million people worldwide were living with chronic hepatitis B (CHB) infection, leading to ~1.1 million deaths annually from liver cirrhosis and cancer. Current treatments for CHB include nucleos(t)ide analogues (NAs), and pegylated interferon (IFN)-α. These treatments are not curative. NA treatments suppress reverse transcription of HBV pregenomic RNA but have no direct effect on covalently closed circular DNA; as a result, peripheral viral loads frequently rebound on NA cessation.[1] Current prophylactic HBV vaccines, based primarily on the HBV small surface antigen (S-HBsAg), induce protective antibody responses in HBV-naïve individuals that can prevent infection but have limited efficacy against pre-existing chronic infection.[2] One potential explanation is that in CHB, high levels of circulating S-HBsAg lead to antigen-specific immune tolerance[3] and exhaustion of S-HBsAg-specific B and T cells.[4] There remains an unmet need for an effective treatment capable of inducing a functional cure for CHB, defined as sustained HBsAg loss and undetectable serum HBV DNA, with or without hepatitis B surface antibody (anti-HBs) seroconversion.[5]

The PreS1 region of the HBV large surface antigen (L-HBsAg) has been proposed as a target antigen for therapeutic HBV vaccination. PreS1 is essential for virus entry into target cells through interactions with the cellular receptor for HBV and HDV (sodium taurocholate co-transporting polypeptide [NTCP]).[6,7] Antibodies against PreS1 can inhibit HBV and HDV infection.[8–10] Furthermore, PreS1-specific B and T cells may be less functionally exhausted in CHB than those recognising S-HBsAg,[11] as circulating levels of L-HBsAg are typically far lower than those of S-HBsAg, with non-infectious subviral particles rich in S-HBsAg exceeding infectious Dane particles by several orders of magnitude during chronic infection.[12] Earlier studies have suggested that anti-PreS1 antibody responses are typically weak or absent in patients with CHB, thus presenting an opportunity for therapeutic intervention.[13,14] Indeed, PreS1 seroconversion has been proposed as an early prognostic marker for the resolution of acute infection,

* Corresponding authors. Addresses: CAMS Oxford Institute, Nuffield Department of Medicine, University of Oxford, Oxford, UK Tel: +44 (0)1865 612900 (J.A. McKeating) or SpyBiotech Ltd, 7600 The Quorum, Oxford Business Park North, Oxford, UK Tel: +44 (0)1865 582088 (M.D.J. Dicks).
E-mail addresses: jane.mckeating@ndm.ox.ac.uk (J.A. McKeating), matthew.dicks@spybiotech.com (M.D.J. Dicks).
https://doi.org/10.1016/j.jhepr.2026.101997

and is rarely observed in patients who progress to chronic infection.[14] Wang *et al.*[15] used SpyTag and SpyCatcher protein superglue technology[16] to attach PreS1 to a ferritin nanoparticle. PreS1-displaying nanoparticles neutralised HBV infection *in vitro* and significantly reduced levels of HBsAg and HBV DNA in an adeno-associated virus-based murine model of HBV in both prophylactic and therapeutic settings.[15]

A therapeutic strategy that induces neutralising anti-PreS1 antibodies could limit the spread of infection, inhibit replenishment of viral reservoirs,[17] and target infected cells via antibody-dependent cellular cytotoxicity.[18] However, it is widely accepted that the establishment of T-cell-mediated immune control will be important for successful therapeutic HBV vaccination.[19] HBV-specific CD8$^+$ T-cell responses are critical for the resolution of acute infection[20] but are dysfunctional during chronic disease. Recombinant adenovirus (Ad) vectors are among the most potent inducers of T-cell immunity, particularly CD8$^+$ T-cell responses, in humans.[21,22] Recently, a chimpanzee Ad encoding multiple HBV antigens induced robust viral-specific T-cell responses in healthy volunteers and lower but significant responses in patients with CHB after a single administration.[23] Combining this vaccine with a modified vaccinia virus Ankara (MVA) vector encoding the same HBV antigens in a prime-boost regimen generated stronger T-cell responses in patients with CHB and significantly reduced surface antigen expression in a subset of patients with lower baseline HBsAg.[24] Although humoral immunity was not measured in either of these clinical studies,[23,24] HBs antibody responses were modest in preclinical studies with these viral vector vaccines.[25] Another promising approach currently in phase I trials is TherVacB, a heterologous prime-boost vaccination strategy using particulate HBsAg and HBcAg protein vaccines and an MVA vector encoding the same antigens. This regimen elicits strong humoral and cellular immunity and has been shown to reduce peripheral HBsAg and HBV DNA levels in HBV mouse models.[26] Importantly, TherVacB does not contain PreS1, and HBV mouse models do not fully recapitulate chronic inflammatory disease in humans because they often lack an adaptive immune system or do not support the complete replicative HBV life cycle.

Building on the approaches described above, we created a recombinant Ad vector encoding L-HBsAg and HBcAg and simultaneously decorated the Ad with the PreS1 peptide. HBcAg forms the internal capsid structure of the HBV particle, and HBcAg-specific T cells are more abundant and less functionally exhausted than S-HBsAg-specific T cells in CHB.[27,28] Covalent attachment of PreS1 to the Ad capsid surface was achieved using the DogTag/DogCatcher protein superglue system, which is similar to SpyTag/SpyCatcher but performs more efficiently in capsid loop structures.[29] We previously introduced modular capsid decoration as a strategy to improve the potency and durability of humoral immune responses by attaching target antigens for pathogen neutralisation to the Ad particle surface.[30] Importantly, decorated Ad retained the ability to induce robust CD8$^+$ T-cell immunity against encoded transgene antigens.[30]

In the present study, immunisation of mice with PreS1-decorated Ad induced superior serum responses capable of neutralising HBV compared with those induced by an undecorated Ad encoding L-HBsAg. Decorated Ad particles induced T-cell responses against encoded antigens comparable to those induced by undecorated particles. Our therapeutic vaccine concept, combining PreS1-targeted neutralisation of infectious HBV with induction of cellular immunity using a potent T-cell-inducing Ad-based platform, has the potential to augment current therapeutic strategies against CHB.

Materials and methods

Cloning and production of adenoviral vectors encoding HBV genes

The construction of Ad-DogTag, an E1/E3-deleted (replication-defective) Ad serotype 5 vector with DogTag inserted into hexon hypervariable loop 5 (HVR5) was described previously.[30] Ad-DogTag constructs expressing combinations of S-HBsAg, L-HBsAg, and HBcAg from genotype D, serotype ayw (NC_003977, human codon-optimised) were generated by cloning gene constructs into pENTR4.CMVp and subsequently inserting them into the E1 locus using Invitrogen Gateway site-specific recombination technology. HBV gene cassettes are described in Fig. S1. Recombinant Ad-DogTag vectors were produced, purified, and titred as described previously.[30]

Protein production and purification

DogCatcher-PreS1 was expressed in suspension ExpiCHO-S cells (Thermo Fisher, UK), affinity purified using C-tag affinity resin (Thermo Fisher, UK) on an AKTA chromatography system (GE Healthcare, UK), and dialysed against Tris-buffered saline at pH 7.8. PreS1 without DogCatcher, cloned into pGEX-6P-1, was transformed into BL21DE3 cells (Life Technologies, UK). The GST-PreS1 protein was purified from the *Escherichia coli* pellet using Glutathione Spin columns (Pierce, UK). The PreS1 protein was then separated from GST using PreScission Protease (GE Healthcare, UK), following the manufacturer's on-column cleavage protocol. Purified PreS1 was dialysed against Tris-buffered saline at pH 7.8.

Coupling reactions

For *in vitro* assays, coupling reactions between the DogCatcher-PreS1 protein and Ad-DogTag were performed by co-incubating 1E+10 viral particles with DogCatcher-PreS1 at a range of micromolar concentrations. Reactions were incubated for 16 h at 4 °C in a total volume of 20 µl of sucrose storage buffer (10 mM Tris-HCl, 7.5% w/v sucrose, pH 7.8). Ligand-decorated vector batches for vaccine studies were prepared by scaling up the *in vitro* reactions from 20 µl to 1 ml. Excess ligand was removed using SpectraPor dialysis cassettes with a 1,000-kDa molecular weight cutoff (Spectrum Labs, UK). Dialysis decreased the excess ligand by at least 10-fold, as measured by densitometry on Coomassie-stained SDS-PAGE. Ligand coverage was determined by SDS-PAGE, as described previously.[30]

Mouse immunisation

All mouse procedures were performed in accordance with the terms of the UK Animals (Scientific Procedures) Act (Project

Licence PP5949437) and approved by the Oxford University Ethical Review Body. Female C57BL/6 mice, six per group (aged 6–8 weeks; Envigo, UK), housed in specific pathogen-free environments, were immunised intramuscularly by injection of 50 μl of vaccine formulated in endotoxin-free PBS (Gibco, UK) into each hind limb (100 μl in total per animal). The Ad vector vaccine dose was 1E+8 infectious units (IFU) per animal per immunisation. The DogCatcher-PreS1 protein vaccine dose was 0.2 μg per animal per immunisation, equivalent to the PreS1 antigen dose delivered by Ad:PreS1^{high} vaccination, as calculated from the previously described capsid antigen dose.[30] Protein vaccines were formulated with AddaVax (InvivoGen, USA) at a 1:1 v/v ratio. The endotoxin dose was <3 EU per mouse. Experiments were performed at Biomedical Services, University of Oxford, Oxford, UK.

IgG ELISA

An IgG endpoint ELISA was performed as described previously,[31] with plates coated with 0.5 μg/ml PreS1. Plates were blocked with 10% milk powder in 0.05% PBS–Tween 20. Serum was diluted threefold in 0.05% PBS–Tween 20, with a starting dilution of 1:100. The secondary antibody (STAR117A, Bio-Rad, UK) was diluted 1:5,000, and the signal was developed in *p*-nitrophenyl phosphate solution. The plates were read at 405 nm using an Infinite M Plex (Tecan, UK). Data were analysed using Prism version 10 (Dotmatics, Boston, MA, USA).

Ex vivo IFN-γ ELISpot

Ex vivo IFN-γ ELISpot was performed on freshly isolated splenocytes according to standard protocols, as described previously.[30] To measure antigen-specific responses, cells were restimulated with peptides (Insight Biotechnology, UK) at a final concentration of 5 mg/ml for 18–20 h. CD8$^+$ T-cell responses to GFP were measured using peptide EGFP$_{118\text{-}126}$.[32] CD8$^+$ T-cell responses to L-HBsAg and S-HBsAg were measured using Env$_{190\text{-}197}$.[33] CD8$^+$ T-cell responses to HBcAg were measured using Core$_{93\text{-}100}$.[34] Spot-forming cells were counted using an automated ELISpot reader system (AID Classic, Germany) with software version 7.

Splenocyte stimulation, intracellular cytokine staining, and flow cytometry

Frozen mouse splenocytes were thawed and rested overnight. The next day, the splenocytes were washed, counted, and seeded at 1×10^7 cells/ml in the presence of 1 μl/ml GolgiPlug (BD Biosciences, UK) and a specific stimulation peptide or peptide pool at 1 μg/ml per peptide. To measure T-cell responses to HBV L, a pool of 95 15-mer peptides with an 11-mer overlap was used (ProImmune, UK). To measure T-cell responses to HBV Core, PepMix HBV (Capsid Protein) Ultra (JPT, Germany), which consists of 155 individual 15-mer peptides with an 11-mer overlap, was used. As a positive control, each splenocyte sample was incubated with 1 μl/ml GolgiPlug, 20 ng/ml phorbol 12-myristate 13-acetate (Merck, UK), and 1 μg/ml ionomycin (Merck, UK). After 6 h of incubation at 37 °C with 5% CO_2, the cells were stored at 4 °C overnight. The next day, the cells were stained. See the Supplementary methods for the staining protocol and antibody panel information. Samples were analysed using an Attune NxT Flow Cytometer (Thermo Fisher, UK), and data were analysed using FlowJo version 10.10 (BD Biosciences, UK).

HBV neutralisation assay

Heat-inactivated sera from mouse experiments were pooled and incubated for 1 h at 37 °C with an HBV reporter virus (multiplicity of infection, 600) expressing Gaussia luciferase (rHBV-gLuc). This mixture was then added to HepG2-NTCP cells[7] (see the Supplementary methods for HepG2-NTCP cell preparation). In brief, rHBV-gLuc is a heparin-purified,[35] single-cycle virus that uses authentic HBV entry pathways but is replication-incompetent as a Gaussia luciferase gene has been engineered in place of the viral polymerase,[36] Six days after infection, the level of Gaussia luciferase was measured by mixing 60 μl of cell culture supernatant with an equal volume of coelenterazine substrate (500 μg/ml) (Promega, UK) in a 96-well white plate (Corning, USA). The plate was then immediately read on a FluoStar (BMG Labtech, UK) with 1-s integration.

HDV neutralisation assay

Heat-inactivated sera from mouse experiments were pooled, diluted to 1:125, and incubated for 1 h at 37 °C with HDV (multiplicity of infection, 50 genome copies per cell) before being added to HepG2-NTCP cells. Six days after infection, HDV-infected cells were detected with an anti-HDAg antibody (pan-genotypic [FD5C4]; Kerafast, USA), and nuclei were detected with DAPI (Life Technologies, UK). See the Supplementary methods for HDV and HepG2-NTCP cell preparation.

Preparation of samples from patients with CHB

All patients provided written consent for the collection of blood samples by the King's College Hospital Liver Research Biobank for research purposes (IRAS project 332608; REC reference 23/LO/0708). The research was conducted in accordance with both the Declaration of Helsinki and the Declaration of Istanbul. Collection tubes were inverted six to eight times and then centrifuged at 2,200 × *g* for 15 min. The supernatant was stored at -20 °C in the biobank repository.

Statistical analyses

Statistical analyses were performed using GraphPad Prism version 10 (Dotmatics, Boston, MA, USA). Comparisons between multiple groups were performed using the Kruskal–Wallis with Dunn's test for multiple comparisons. Pairwise comparisons (two groups only) were performed using the Mann–Whitney *U* test. Statistical significance is indicated as follows: *p <0.05, **p <0.01, ***p <0.001, and ****p <0.0001. Non-significant comparisons are not shown. Where only certain statistical comparisons have been made, this is described in the figure legend.

Results

PreS1 antibody titres are rarely observed in patients with CHB

Previous studies have reported that antibodies against PreS1 can inhibit HBV particle binding to the NTCP receptor and

neutralise infection.[8] However, only a small percentage (5–7.5%) of individuals with CHB develop anti-PreS1 responses.[13,14] To confirm previous observations, we performed anti-PreS1 endpoint ELISA assays with sera from 61 treatment-naïve patients with CHB, with each of the five major HBV genotypes (A–E) represented in the cohort (Fig. 1A). Consistent with previous reports, only six of 61 (9.8%) patients with CHB had detectable anti-PreS1 antibodies above background levels (Fig. 1B), with genotypes A, B, and E represented among all the responders, despite using a genotype D serotype ayw PreS1 in the assay. Alignment of the PreS1 amino acid sequences from genotypes A to E shows a high degree of conservation between the genotypes (Fig. 1C), with 100% identity in the NTCP receptor-binding domain.[6,9] These data suggest that anti-PreS1 antibodies raised against one genotype may neutralise other HBV genotypes. In summary, our data confirm that anti-PreS1 antibody titres are low in CHB, suggesting a therapeutic opportunity to induce, restore, or rescue this response in chronic disease.

Extensive coverage of recombinant Ad particles with PreS1 can be achieved without reducing vector infectivity

We used the DogTag/DogCatcher protein superglue to decorate recombinant Ad vector particles with the PreS1 antigen. DogTag (23 amino acids) was genetically inserted into surface-exposed loops on the Ad capsid (hexon),[30] and DogCatcher (15 kDa) was fused to the N terminus of PreS1 (genotype D, serotype ayw) for expression as a recombinant protein. Upon co-incubation, hexon-DogTag and DogCatcher-PreS1 formed a spontaneous isopeptide bond, resulting in covalent attachment of PreS1 to Ad particles (Fig. 2A). Increasing concentrations of DogCatcher-PreS1 resulted in a dose-dependent increase in attachment up to a maximum of 70% of virion-associated hexon proteins (Fig. 2B,C). Adding additional ligand did not increase coverage beyond 70%, presumably because of steric hindrance. Nonetheless, because there are 720 copies of hexon per virion, 70% coverage corresponds to ~500 copies of PreS1 per particle. Importantly, even at the

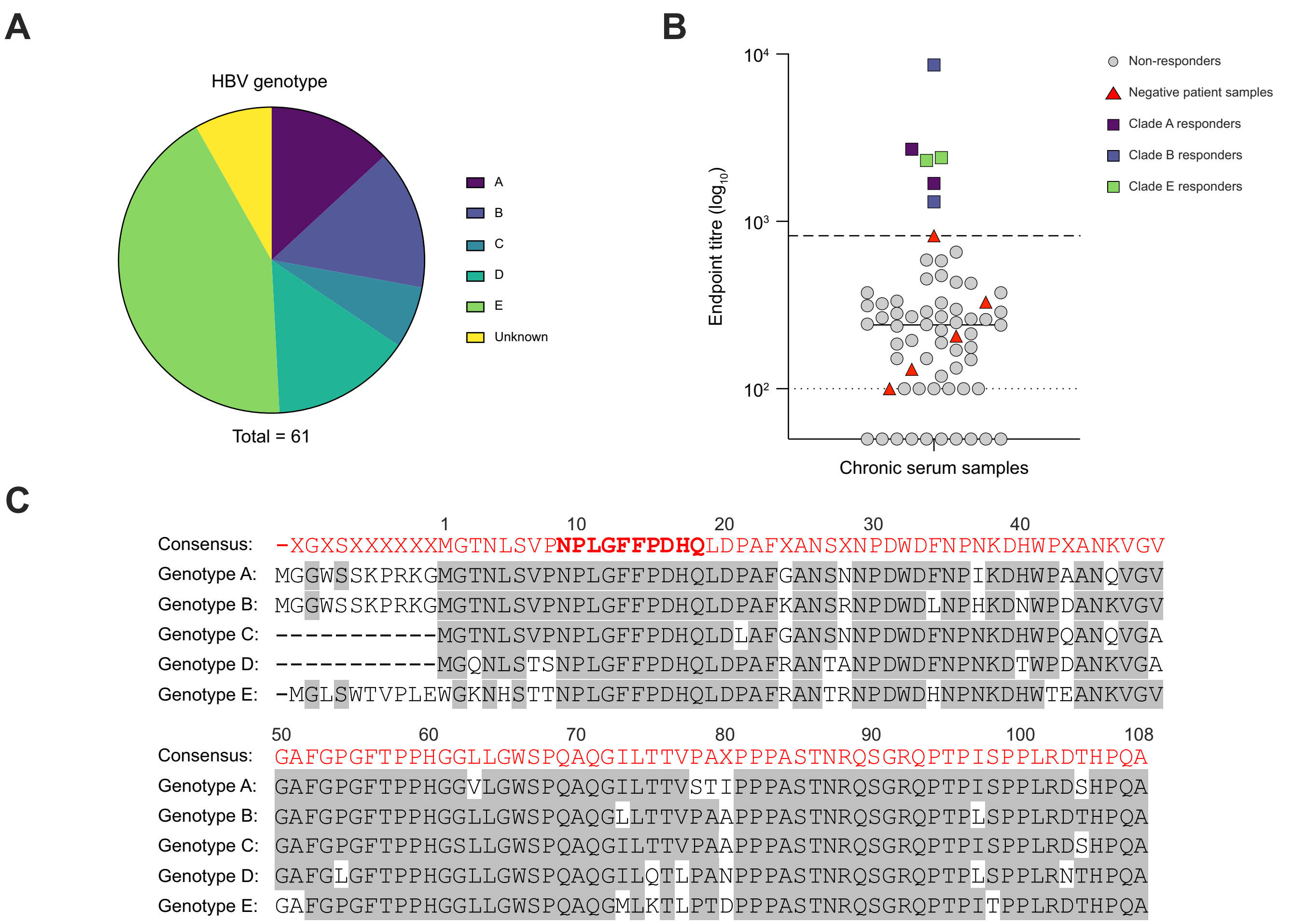

Fig. 1. Anti-PreS1 antibody responses are rarely observed in individuals with CHB. (A) Distribution of genotypes found in CHB samples tested in (B). (B) Levels of anti-PreS1 antibodies were measured by endpoint ELISA in serum from 61 patients with CHB and 5 HBV-negative patients. Red triangles, negative patients; grey circles, patients with CHB with no anti-PreS1 antibodies (non-responders); purple, blue, and green squares, patients with CHB with anti-PreS1 antibodies (responders). The different colours correspond to the different patient genotypes using the same colour scheme as in (A). Lower dotted line, LOD; interpolated values below the LOD were reassigned the value of the LOD, and uninterpolatable values were assigned an arbitrary value of 50 to plot them on the graph. Higher dashed line, upper limit of negative patients. (C) Alignment of the PreS1 amino acid sequence from genotypes A–E. Amino acids highlighted in grey match the consensus sequence in red. The sequence in bold red from amino acids 9 to 18 (counting based on genotype D, serotype ayw) is the NTCP-binding domain.[6,9] CHB, chronic hepatitis B; LOD, limit of detection; NTCP, sodium taurocholate co-transporting polypeptide.

highest levels of particle coverage, we observed no reduction in the ability of PreS1-decorated Ad vectors to transduce HEK293 cells *in vitro* compared with an undecorated Ad (Fig. 2C).

PreS1-decorated Ad elicits anti-PreS1 serum antibody responses

To assess the immunogenicity of PreS1-decorated Ad, we immunised C57BL/6 mice with GFP-encoding Ad vectors, either undecorated or with low (30%, Ad(GFP):PreS1^{low}) or high (70%, Ad(GFP):PreS1^{high}) PreS1 capsid coverage. A further group of animals was immunised with the DogCatcher-PreS1 recombinant protein alone (at an equivalent PreS1 protein dose to Ad[GFP]:PreS1^{high}-vaccinated animals), formulated in a squalene-based adjuvant (AddaVax) (Fig. 3A). Three weeks after a single vaccine dose, IgG antibody responses against PreS1 were generated in most animals vaccinated with PreS1-decorated Ad (6/6 Ad[GFP]:PreS1^{high}, 4/6 Ad[GFP]:PreS1^{low}). By contrast, no detectable responses were observed after vaccination with the DogCatcher-PreS1 protein in adjuvant (Fig. 3B). Two weeks after a homologous boost, anti-PreS1 IgG responses further increased in animals vaccinated with PreS1-decorated Ad, with robust responses achieved in all animals. By contrast, only four of six animals vaccinated with the DogCatcher-PreS1 protein achieved detectable responses after boost (Fig. 3B). As expected, no anti-PreS1 responses were generated in animals vaccinated with the undecorated Ad(GFP) vector. In the same experiment, we assessed the impact of Ad capsid decoration on cellular immune responses against GFP. No differences in the magnitude of splenic CD8$^+$ T-cell responses were observed between PreS1-decorated and undecorated Ad (Fig. 3C). These data demonstrate that PreS1 capsid decoration does not impair cellular immune responses against the encoded transgene.

PreS1-decorated Ad encoding HBV antigens induces antiviral humoral and cellular immunity

In parallel, Ad vectors encoding various arrangements of L-HBsAg, S-HBsAg, and HBcAg were designed and produced (Fig. S1A). Expression of HBV antigens from Ad-transduced HEK293 cells was confirmed by Western blotting (Fig. S1B). Multiple L-HBsAg and S-HBsAg bands were detected, likely as a result of different glycosylation patterns.[37] To enable the expression of multiple antigens to be driven by a single promoter, we used the 2A sequence from foot-and-mouth disease

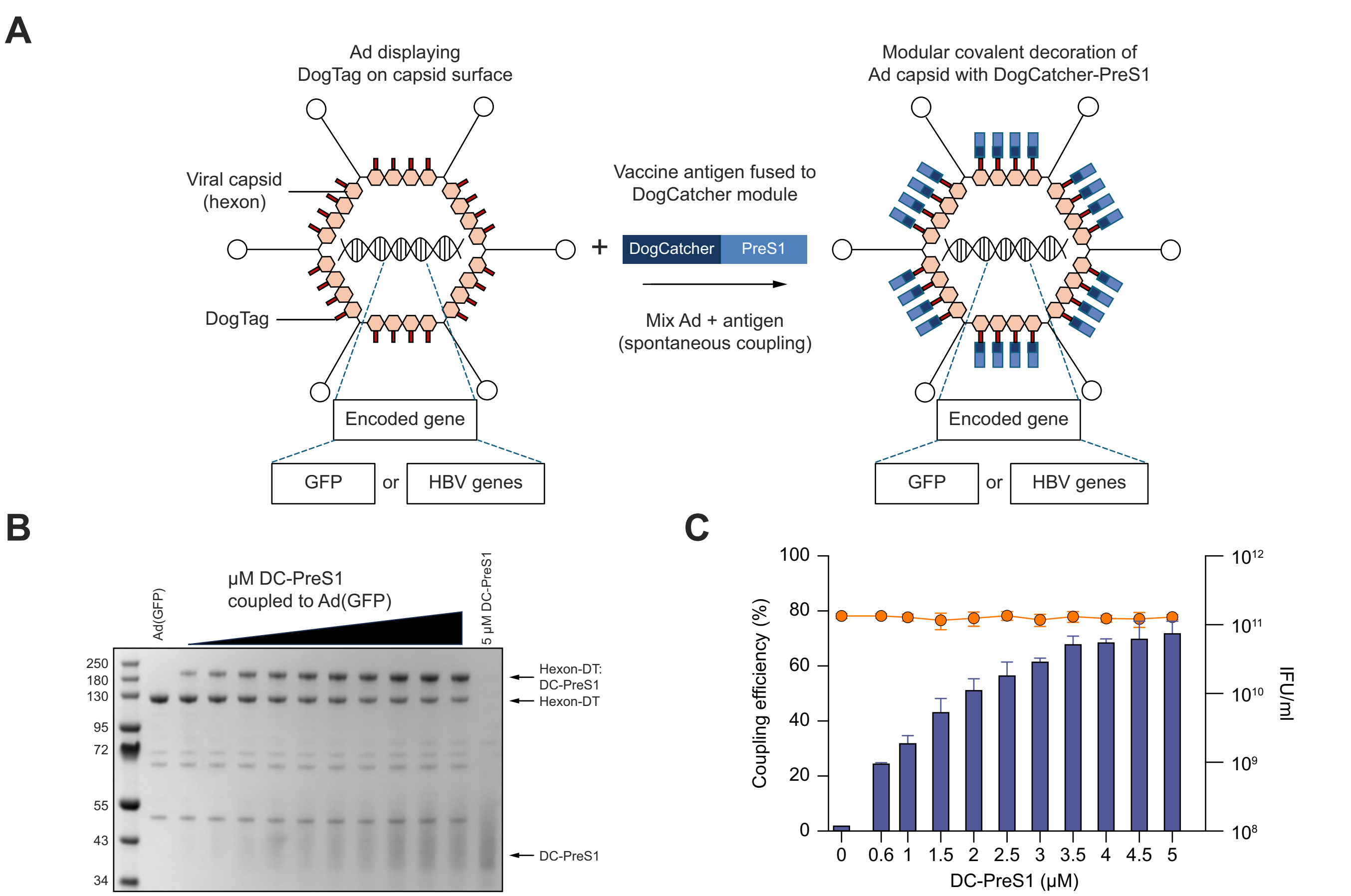

Fig. 2. HBV PreS1 can be efficiently coupled to the Ad hexon protein with no loss of Ad infectivity. (A) Display of the DogCatcher-fused PreS1 peptide on the surface of the Ad capsid achieved via covalent attachment to DogTag inserted into hexon. (B) Representative SDS-PAGE and Coomassie blue staining analysis. (C) Coupling efficiency (% hexon coverage; blue bars) and infectivity (IFU/ml; orange circles) of Ad-DogTag virions incubated with an increasing concentration of the DogCatcher-PreS1 protein (0.6–5 mM). Samples were analysed in duplicate, and the data show mean ± SD of triplicate independent repeats. Ad, adenovirus; IFU, infectious units.

virus to promote ribosome skipping.[38] For Ad($L_{2A}C$), incomplete 2A 'cleavage' between L and C proteins was observed, leading to the production of a fusion protein.

An immunogenicity study using C57BL/6 mice was conducted to compare Ad vectors encoding HBcAg and S-HBsAg antigens ($C_{2A}S$) with those encoding L-HBsAg (L). The Ad($C_{2A}S$) and Ad(L) vectors were either undecorated or decorated with PreS1 at ~30% capsid coverage (comparable to PreS1^{low} in Fig. 3). Immunisation was performed using the same homologous prime-boost schedule as described previously in Fig. 3A. Serum anti-PreS1 IgG responses were measured by ELISA at Days 20 and 35 (Fig. 4A). As expected, Ad vectors only elicited detectable PreS1 antibody responses if they encoded L or were decorated with PreS1. By Day 35, both PreS1-decorated Ads induced higher PreS1 antibody titres than undecorated Ad(L), with the highest titres induced by PreS1-decorated Ad encoding L (Ad(L):PreS1) (Fig. 4A). $CD8^+$ T-cell responses in vaccinated animals were assessed by spleen IFN-γ ELISpot (Fig. 4B). Robust $CD8^+$ T-cell responses against L/S were detected in all animals, with Ad vectors encoding L eliciting stronger responses than vectors encoding $C_{2A}S$. PreS1 decoration had no effect on the magnitude of T-cell responses (Fig. 4B). Similarly, strong $CD8^+$ T-cell responses against C were elicited in both groups vaccinated with Ad($C_{2A}S$), with response magnitudes comparable between PreS1-decorated and undecorated vectors (Fig. 4B). Antigen-specific T-cell cytokine responses were assessed by intracellular cytokine staining using pools of overlapping peptides covering full-length L-HBsAg (L) and HBcAg (C) (Fig. 4C). The gating strategy and controls are shown in Figs. S2 and S3. In agreement with the IFN-γ ELISpot data, PreS1 decoration had no effect on the magnitudes of $CD8^+$ T-cell cytokine responses, and Ad(L) induced higher L-specific IFN-γ and TNF-α responses than Ad($C_{2A}S$) (Fig. 4C and Fig. S3). L- and C-specific IFN-γ^+CD4^+ T-cell responses were also detected, albeit at slightly lower frequencies than the IFN-γ^+CD8^+ responses (Fig. 4C). However, $CD4^+$ IL-2 and TNF-α responses were minimal (Fig. S3).

As the PreS1 region of L-HBsAg is essential for HBV entry into cells, we hypothesised that anti-PreS1 antibodies induced by these vaccines would neutralise virus infections. We established an *in vitro* HBV neutralisation assay using Gaussia luciferase-expressing reporter HBV (rHBV-gLuc) for *de novo* infection of hepatoma cells (Fig. 4D). This virus uses authentic HBV entry mechanisms, including PreS1 engagement with NTCP, to infect cells. Incubation of rHBV-gLuc with either an anti-PreS1 monoclonal antibody or the peptide entry inhibitor myrcludex B (bulevirtide; Hepcludex™) successfully inhibited the infection of HepG2-NTCP cells (Fig. 4E). Myrcludex B is a myristolated lipopeptide comprising 47aa (2-48) from the PreS1 region, which binds to NTCP, thereby blocking HBV and HDV cell entry. Serum (at Day 35) from mice immunised with undecorated Ad($C_{2A}S$) or Ad(L) showed only weak HBV neutralising activity (2.7- and 3.8-fold inhibition, respectively,

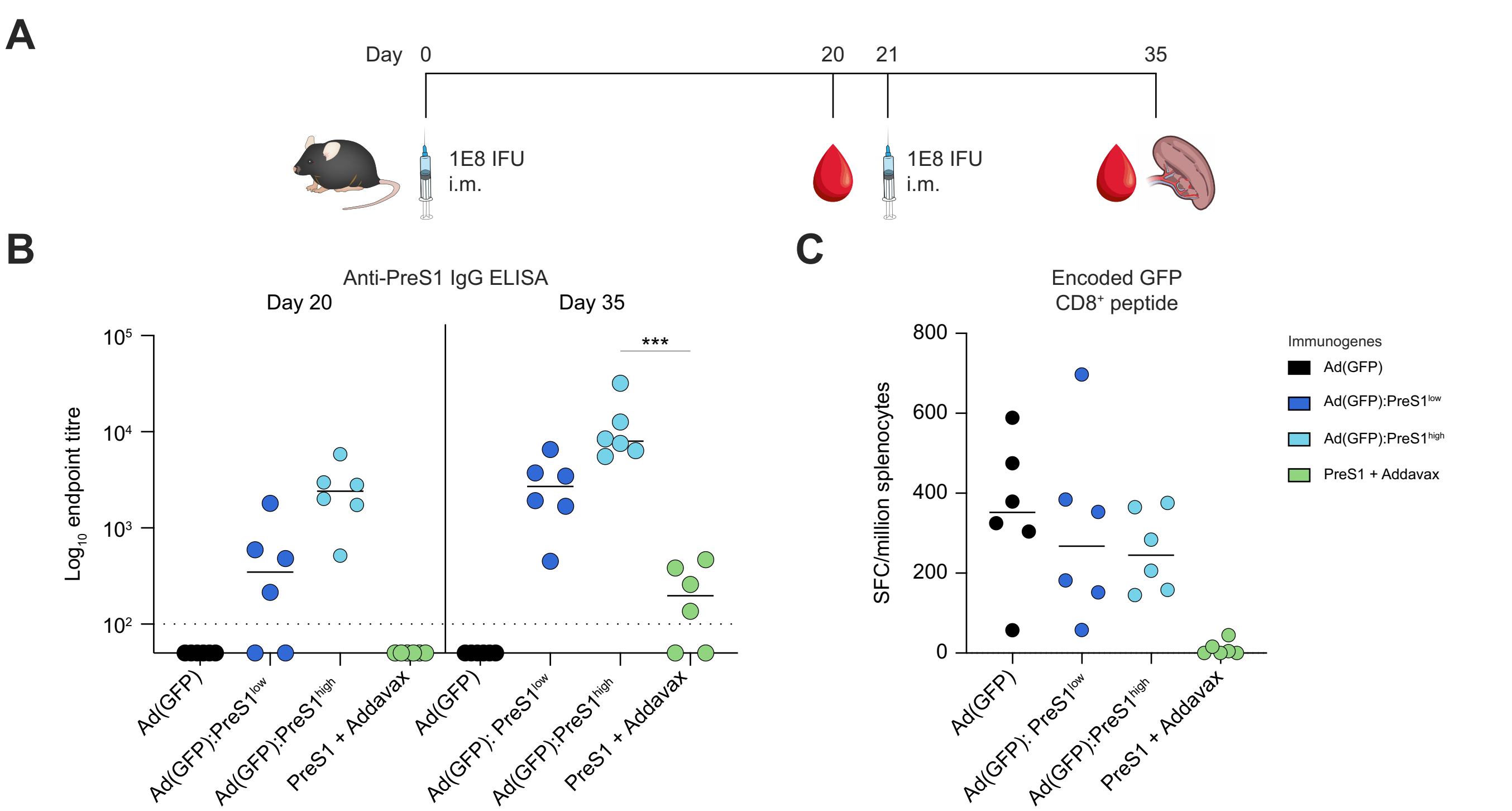

Fig. 3. Anti-PreS1 antibodies are generated by capsid display of PreS1 peptide while maintaining encoded antigen $CD8^+$ T-cell responses. (A) Schematic immunisation schedule. C57BL/6 mice (six per group) were immunised intramuscularly (i.m.) in homologous prime-boost regimens with 1E+8 IFU per mouse of undecorated Ad(GFP), Ad(GFP) with either low (30%, PreS1^{low}) or high (70%, PreS1^{high}) PreS1 capsid coverage, or the DC-PreS1 protein in AddaVax (0.2 μg). (B) Serum IgG antibody responses to PreS1 at Days 20 and 35 measured by endpoint ELISA. Dotted line represents the limit of detection. Uninterpolated samples were given an arbitrary value of 50. Titres at Day 35 in the PreS1-containing vaccine groups were statistically compared. (C) IFN-γ ELISpot responses (SFC/million splenocytes) in spleens at Day 35 against a GFP $CD8^+$ T-cell epitope peptide, DTLVNRIEL. The Ad-vaccinated groups were statistically compared. For all graphs, median responses are shown, and statistical analyses were determined using the Kruskal–Wallis with Dunn's test for multiple comparisons, ***p <0.001; non-significant comparisons are not shown. IFU, infectious units; SFC, spot-forming cells.

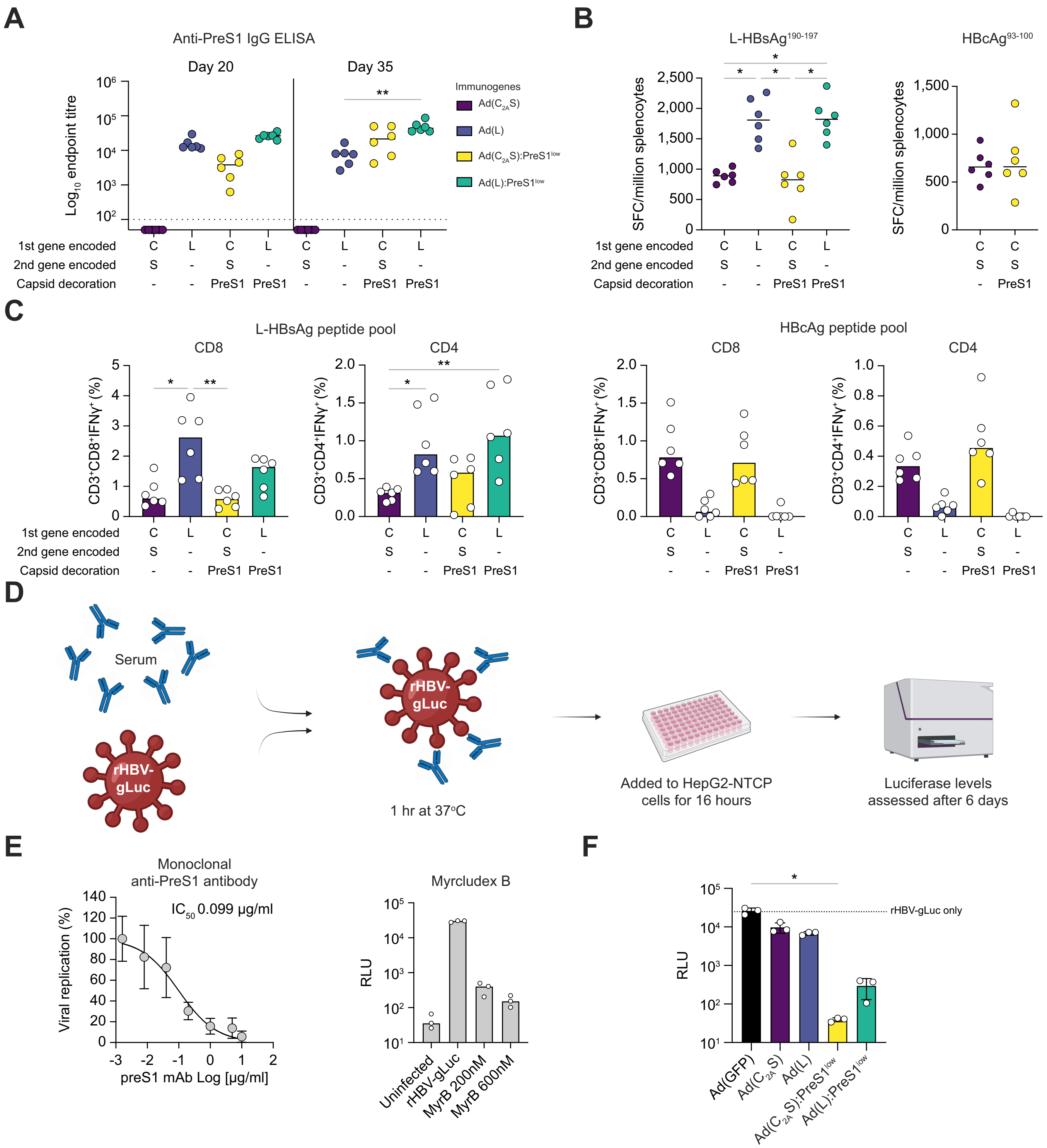

Fig. 4. Optimal anti-PreS1 antibody responses and anti-L T-cell responses produced by vectors decorated with PreS1 and encoding L. C57BL/6 mice (six per group) were immunised intramuscularly (i.m.) with Ad vectors encoding HBcAg and S-HBsAg or L-HBsAg with or without PreS1 peptide decoration in homologous prime-boost regimens. Vaccine immunogens in each group are detailed in (A) and below each graph. (A) Serum IgG antibody responses to PreS1 at Days 20 and 35 measured by endpoint ELISA. Dotted line represents the limit of detection. Uninterpolated samples were given an arbitrary value of 50. Titres at Day 35 in the PreS1-containing vaccine groups (encoded or surface displayed) were statistically compared. (B) IFN-γ ELISpot responses (SFC/million splenocytes) in spleens at Day 35 against an L-HBsAg $CD8^+$ T-cell epitope peptide (VWLSVIWM) or a HBcAg $CD8^+$ T-cell epitope peptide (MGLKFRQL). (C) Percentages of IFN-γ-producing $CD3^+$ $CD8^+$ T cells and $CD3^+$ $CD4^+$ T cells at Day 35 responding to L-HBsAg and HBcAg peptide pools. For the HBcAg peptide pools, only groups encoding HBcAg were statistically compared to assess the effect of PreS1 decoration on anti-Core $CD8^+$ T-cell responses. (D) Schematic describing the HBV neutralisation assay. (E) Neutralisation of rHBV-gLuc infection using either a dilution series of a mouse monoclonal anti-PreS1 antibody or two concentrations of myrcludex B (bulevirtide). (F) Neutralisation of rHBV-gLuc with 1:250-diluted heat-inactivated pooled serum from mice immunised with Ad vectors encoding HBV genes with or without PreS1 capsid decoration. Experiments were performed in technical triplicate with three independent repeats. For (A)–(C) and (F), median responses are shown; for (E) and

relative to control Ad(GFP) at a 1:250 serum dilution), whereas PreS1-decorated Ad(C_{2A}S):PreS1 and Ad(L):PreS1 elicited serum responses that neutralised HBV (662- and 89-fold inhibition, respectively, at a 1:250 dilution) (Fig. 4F). These data demonstrate that PreS1 capsid decoration is required to induce serum neutralising antibodies.

Optimising anti-HBV immunity with PreS1-decorated Ad

To further improve immunogenicity, Ad vectors encoding L, L_{2A}C, and C_{2A}L were decorated with PreS1 at 70% capsid coverage (comparable to PreS1^{high} in Fig. 3), and C57BL/6 mice were immunised using the same dosing regimen. Robust anti-PreS1 IgG ELISA titres were achieved in all three groups and were comparable between groups (Fig. 5A). $CD8^+$ T-cell responses against an L/S epitope, as assessed by IFN-γ ELISpot, were comparable between all three groups demonstrating that the addition of C and the order of L and C antigens had a minimal effect on the magnitude of the T-cell responses (Fig. 5B). Similar responses were noted against the C epitope between the Ad(L_{2A}C):PreS1- and Ad(C_{2A}L):PreS1-vaccinated groups (Fig. 5B). $CD8^+$ and $CD4^+$ T-cell cytokine responses against L were comparable between all three groups (Fig. 5C and Fig. S4). $CD8^+$ and $CD4^+$ T-cell cytokine responses against the C peptide pool were comparable between the Ad(L_{2A}C):PreS1- and Ad(C_{2A}L):PreS1-vaccinated groups, although there was a non-significant trend towards higher IL-2^+CD8^+ T cells in Ad(L_{2A}C):PreS1-vaccinated animals (Fig. S4).

Serum HBV neutralisation was assessed as described previously. Mice vaccinated with Ad(L):PreS1, Ad(L_{2A}C):PreS1, and Ad(C_{2A}L):PreS1 showed neutralising activity (Fig. 5D). Unlike the previous experiment, neutralisation was maintained upon serial dilution of the serum up to at least 1:16,000, suggesting a more robust response. ID_{50} values were 120,956 for Ad(L):PreS1, 264,763 for Ad(L_{2A}C):PreS1, and 119,051 for Ad(C_{2A}L):PreS1. The strongest serum neutralisation was observed in the Ad(L_{2A}C):PreS1-vaccinated group, showing a statistically significant difference in neutralisation compared with the control group as determined by AUC analysis (Fig. 5D).

PreS1-decorated Ad achieves serum neutralisation of HDV

HDV is a small, defective RNA virus that can use the HBV-encoded surface glycoproteins to assemble infectious particles[39] and frequently co-infects with HBV. Myrcludex B has been shown to induce sustained virologic and biochemical responses in chronic HDV[40] and is approved in the EU for the treatment of HDV-associated compensated liver disease. Given that both HDV and HBV are reliant on PreS1 engagement with NTCP to infect cells, we hypothesised that vaccine-induced anti-PreS1 antibodies should neutralise HDV infection of HepG2-NTCP cells. To test this, we incubated HDV with pooled serum from mice immunised with Ad(L_{2A}C):PreS1 (from the experiment shown in Fig. 5). In parallel, we incubated HDV with an anti-PreS1 monoclonal antibody and myrcludex B as controls. Complexes were added to HepG2-NTCP cells, and HDV infection was assessed by immunostaining for the HDV antigen 6 days after infection (Fig. 6A). We observed a marked reduction in HDV-infected cells after exposure to serum from mice immunised with Ad(HBV L_{2A}C):PreS1 (Fig. 6B). This phenocopied the results observed with the controls anti-PreS1 (10 μg/ml) and myrcludexB (200 nM). These data suggest that PreS1-decorated Ad may have additional utility in chronic HBV/HDV co-infection, given the homologous cellular entry mechanisms shared by these two pathogens.

Discussion

In this study, we describe a novel therapeutic vaccination strategy that induces neutralising antibody responses and potent T-cell immunogenicity against HBV in a single platform using a PreS1-decorated Ad vector encoding multiple HBV antigens. PreS1 is required for HBV and HDV entry into cells;[8] hence, PreS1-targeted neutralising antibodies could prevent *de novo* infection, limiting the spread of infection and associated intrahepatic inflammation.[41] We confirmed that anti-PreS1 immunity is limited or absent in CHB (Fig. 1B), presenting an opportunity for therapeutic intervention.

Displaying peptide and protein antigens on nanoparticles is an effective strategy to improve antigen-specific antibody titres following vaccination.[42] Here, we used the DogTag/DogCatcher protein superglue to decorate recombinant Ad particles with PreS1 (Fig. 2). PreS1-decorated Ad achieved significantly higher anti-PreS1 IgG titres than the recombinant DogCatcher-PreS1 protein in adjuvant (Fig. 3B) or a conventional undecorated Ad encoding L-HBsAg (Fig. 4A). Crucially, PreS1-decoration was essential for the induction of neutralising activity (Fig. 4F), and highly decorated particles induced a potent response (Fig. 5D, ID_{50} >10^5). A key advantage of PreS1-decorated Ad over protein nanoparticle and virus-like-particle technologies for therapeutic vaccination is that Ad vector vaccines induce potent T-cell immunity, particularly $CD8^+$ T-cell responses, against antigens encoded in the vector genome. PreS1-decorated Ad induced robust $CD8^+$ and $CD4^+$ T-cell responses against encoded HBV antigens, specifically L-HBsAg and HBcAg (Figs. 4 and 5). Bivalent cassettes induced T-cell responses comparable to those of a monovalent cassette expressing only L-HBsAg, indicating that the co-expression of HBcAg did not lower responses against L-HBsAg through antigenic competition.

Despite achieving promising immunogenicity in HBV-naïve mice, it remains to be established whether our strategy can overcome immune tolerance in CHB. Therapeutic strategies using recombinant viral vectors (including Ad) and PreS1-decorated nanoparticles have demonstrated encouraging immunogenicity in immune-tolerant HBV-carrier mouse model[11,15,26] and early-stage clinical studies in patients with CHB.[23] Commonly used immunocompetent murine HBV models, including AAV-HBV and HBV- or HBsAg-transgenic mice, do not support viral spread, creating specific challenges in demonstrating the added benefit of combining entry

(G), mean values with SD are shown. Statistical analyses were performed on data in (A)–(C) and (F) using the Kruskal–Wallis with Dunn's test for multiple comparisons, where more than one comparison was made, and the Mann–Whitney *U* test for a single comparison, *p <0.05; **p <0.01; ***p <0.001; non-significant comparisons are not shown. Ad, adenovirus; L-HBsAg, HBV large surface antigen; RLU, relative light units; SFC, spot-forming cells; S-HBsAg, HBV small surface antigen.

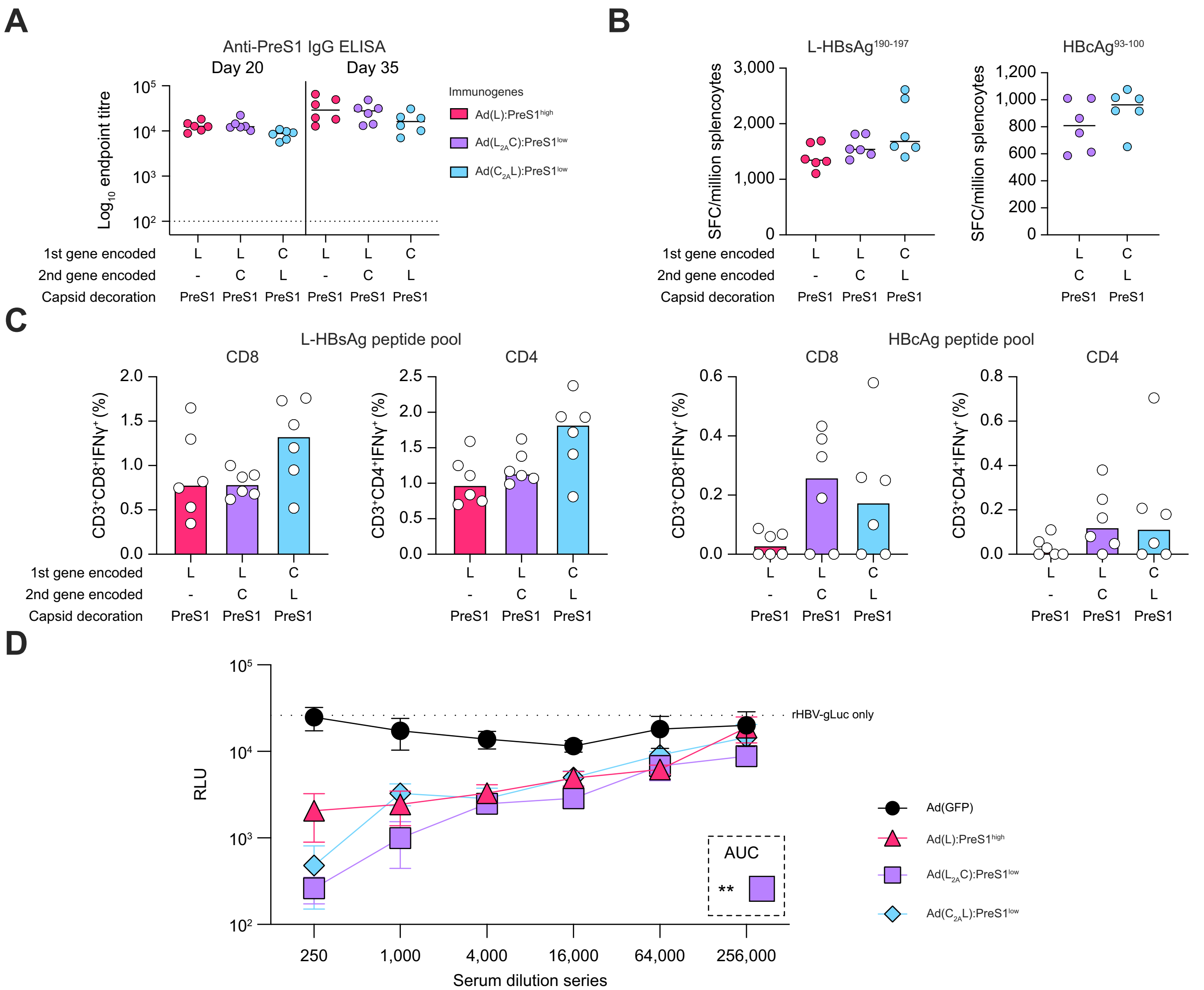

Fig. 5. High levels of PreS1 on the Ad capsid surface induce potent HBV neutralisation. C57BL/6 mice (six per group) were immunised intramuscularly (i.m.) with PreS1-decorated Ad encoding L-HBsAg or L-HBsAg and HBcAg in homologous prime-boost regimens. Immunogens used are detailed in (A) and below each graph. (A) Serum IgG antibody responses to PreS1 at Days 20 and 35 measured by endpoint ELISA. Dotted line represents the limit of detection. The Day 35 groups were statistically compared. (B) IFN-γ ELISpot responses (SFC/million splenocytes) in spleens at Day 35 against an L-HBsAg CD8+ T-cell epitope peptide (VWLSVIWM) or a HBcAg CD8+ T-cell epitope peptide (MGLKFRQL). (C) Percentages of IFN-γ-producing CD3+ CD8+ T cells and CD3+ CD4+ T cells at Day 35 responding to L-HBsAg and HBcAg peptide pools. For the HBcAg peptide pools, only groups encoding HBcAg were statistically compared to assess the effect of PreS1 decoration on anti-Core CD8+ T-cell responses. (D) Neutralisation of rHBV-gLuc with heat-inactivated pooled serum from immunised mice. Experiments were performed in technical triplicate with three independent repeats. AUC was determined for all groups and statistically compared with Ad(GFP). For (A)–(C), median responses are shown; for (D), mean values with SD are shown. Statistical analyses were performed on data in (A)–(C) and AUC of (D) using the Kruskal–Wallis with Dunn's test for multiple comparisons, where more than one comparison was made, and the Mann–Whitney *U* test for a single comparison; non-significant comparisons are not shown. Ad, adenovirus; L-HBsAg, HBV large surface antigen; SFC, spot-forming cells.

inhibition with T-cell induction in our platform. Human liver chimeric models support natural HBV infection and replication but typically lack a functional immune system. The recently described HIS-HUHEP model, which engrafts a functional human immune system into a liver chimeric mouse, represents an elegant approach for modelling CHB infection and may provide a means to fully assess the potential of our platform in the future.[43]

Establishing a functional cure for CHB will almost certainly require a combinatorial approach using multiple therapeutic components targeting different stages of the viral life cycle. Feld *et al.*[44] highlighted three principal strategies: (i) inhibition of viral replication to prevent further spread of infection (*e.g.* entry or capsid inhibitors), (ii) antigen reduction to alleviate immune tolerance (*e.g.* siRNAs), and (iii) immune modulation to re-establish anti-HBV immunity (*e.g.* checkpoint inhibitors and therapeutic vaccines). PreS1-decorated Ad addresses two of these key aspects and could contribute towards the development of a functional cure in combination with translation inhibitors, checkpoint inhibitors, and/or complementary

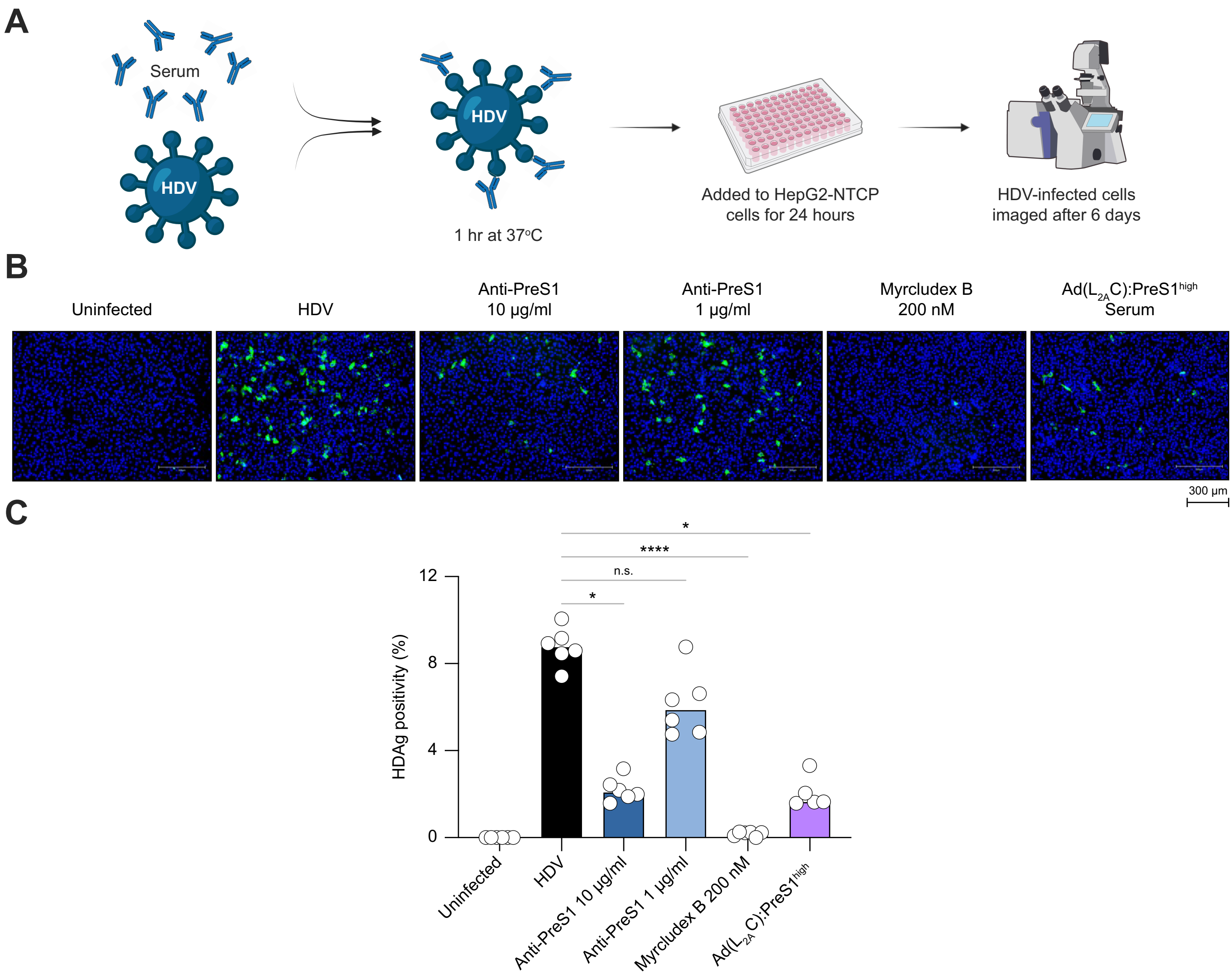

Fig. 6. Serum from mice immunised with PreS1-decorated Ad neutralizes HDV. (A) Schematic of the HDV neutralisation assay. (B) Images of HepG2-NTCP cells either uninfected or infected with HDV in the presence or absence of anti-PreS1 antibodies, myrcludex B, or serum from mice immunised with Ad(L_{2A}C):PreS1 (from the experiment shown in Fig. 5). Representative images of infected wells are shown. Scale bar = 300 µm. (C) Quantification of data shown in (B). The number of HDV-infected, GFP-positive cells relative to the total number of cells (determined by DAPI staining) were determined from two images of three independent wells for each condition. Median responses are shown, and statistical analyses were determined using the Kruskal–Wallis with Dunn's test for multiple comparisons, comparing all test conditions to the HDV-infected control, *p <0.05, ***p <0.001, n.s., non-significant. Ad, adenovirus; NTCP, sodium taurocholate co-transporting polypeptide.

vaccination strategies (*e.g.* heterologous prime-boost regimens). Several combination approaches are currently being assessed in clinical trials. A PreS-containing HBsAg virus-like-particle vaccine (BRII-179) (comprising L-, M-, and S-HBsAg isoforms) co-administered with an siRNA (elebsiran) to lower circulating antigen levels and reduce immune tolerance effectively re-established anti-HBV immunity, including anti-HBs antibodies and PreS1/PreS2-specific $CD4^+$ T-cell responses.[45] The authors hypothesised that anti-HBV neutralising antibodies and PreS1- and PreS2-specific CD4+ T cells may have helped restrict *de novo* infection and re-establish virological control in this patient.[45] BRII-179, a recombinant protein vaccine, did not induce HBV-specific $CD8^+$ T-cell responses. Inducing $CD8^+$ T-cell immunity in this context, perhaps by adding viral vectors, could further improve the virological response to vaccination. In another study, a recombinant Ad vector encoding multiple HBV antigens, ChAdOx1-HBV, was combined with a poxvirus vector, MVA-HBV, in a heterologous prime-boost regimen (VTP-300).[24] The combination with a checkpoint inhibitor (low-dose nivolumab) administered at the time of the MVA boost led to an enhanced reduction in HBsAg.[24] Combining VTP-300 with an siRNA (imdusiran) also led to improved HBsAg reduction compared with siRNA treatment alone in virally suppressed patients.[46]

By targeting PreS1, our approach could provide additional benefits in the context of HBV/HDV co-infection. As expected, serum from mice vaccinated with PreS1-decorated Ad inhibited HDV infection (Fig. 6). HDV/HBV co-infection occurs in approximately 5% of patients with CHB and is considered the most severe form of viral hepatitis. Inducing neutralising antibodies through PreS1 vaccination could serve as an attractive

alternative or complementary approach to treatments involving currently licensed PreS1-based peptide inhibitors, such as bulevirtide. Although there has been no direct head-to-head comparison, anti-HBs monoclonal antibodies have arguably achieved more substantial declines in HDV RNA compared with bulevirtide monotherapy,[47] perhaps through the additional contribution of Fc-receptor mediated effector functions.[48] In a separate study, bulevirtide combined with pegylated IFN-α was more effective in reducing HDV DNA to undetectable levels than bulevirtide monotherapy,[49] further supporting the combination of entry inhibition with immune modulation. Immunological memory acquired through PreS1 vaccination could ultimately provide finite-duration treatments for HDV. Bulevirtide currently requires daily subcutaneous administration, and relapses frequently occur upon withdrawal.[50]

The ability of PreS1-decorated Ad to combine the induction of potent HBV/HDV neutralising humoral immunity with strong cellular immune responses in a single platform differentiates our technology from current state-of-the-art approaches. Conventional viral vector vaccines, including recombinant Ads, typically do not induce strong vaccine antigen-specific B-cell responses. Therefore, many viral vector-based therapeutic vaccine studies have neglected to target (or even assess) the induction of humoral immunity. Conversely, protein-in-adjuvant vaccines may lack activity in CHB because of weak or absent $CD8^+$ T-cell induction. Combining PreS1-targeted entry inhibition with robust anti-HBV/HDV T-cell induction offers a promising strategy to improve therapeutic outcomes in chronic HBV and HDV infection.

Affiliations

[1]*SpyBiotech Ltd, Oxford, UK;* [2]*Institute of Liver Studies, King's College Hospital, London, UK;* [3]*Nuffield Department of Medicine, University of Oxford, Oxford, UK;* [4]*CAMS Oxford Institute, Nuffield Department of Medicine, University of Oxford, Oxford, UK;* [5]*The Jenner Institute, University of Oxford, Old Road Campus Research Building, Oxford, UK*

Abbreviations

Ad, adenovirus; CHB, chronic hepatitis B; L-HBsAg, HBV large surface antigen; HBs, hepatitis B surface; IFU, infectious units; MVA, modified vaccinia virus Ankara; NA, nucleos(t)ide analogue; NTCP, sodium taurocholate co-transporting polypeptide; S-HBsAg, HBV small surface antigen.

Financial support

Research in the McKeating laboratory is funded by the Wellcome Trust Discovery Award (225198/Z/22/Z) and the Chinese Academy of Medical Sciences (CAMS) Innovation Fund for Medical Science (CIFMS) (2024-I2M-2-001-1). PACW was supported by the Chinese Academy of Medical Sciences (CAMS) Innovation Fund for Medical Science (2024-I2M-2-001-1).

Conflicts of interest

RAR, LMR, BGC, and MDJD are employees of SpyBiotech Ltd. SB is CSO and co-founder of SpyBiotech Ltd. The remaining authors declare no conflicts of interest that pertain to this work.

Please refer to the accompanying ICMJE disclosure forms for further details.

Authors' contributions

Conceptualisation: RAR, JL, JMH, LMR, PACW, SB, JAM, MDJD. Methodology: RAR, JL, JMH, ST, LMR, BGC, PACW, JAM, MDJD. Investigation: RAR, JL, JMH, ST, LMR, BGC, PACW. Visualisation: RAR, JL, JMH, ST, PACW, MDJD. Resources: JL, IC, KA.

Writing—original draft: RAR, MDJD. Writing—review & editing: RAR, JL, JMH, ST, JAM, MDJD.

Data availability

Data have not been deposited publicly but can be made available upon reasonable request to the corresponding authors.

Acknowledgements

The authors would like to thank Rosa D'Agostino, Laura Bilbé, Lesley Bowman, Jeanette Wagener, David Bitto, Sophie Porret, Antonia Hook, Laura Silva-Reyes, and Jakub Kopycinski for helpful advice and assistance. We thank the staff at Biomedical Services, University of Oxford, for animal husbandry and technical assistance. We thank Stephan Urban (University of Heidelberg) for supplying HepG2-NTCP cells and myrcludex B and Jochen Wettengel and Ulrike Protzer (Technical University of Munich) for the purified HB-Luciferase reporter virus.

Supplementary data

Supplementary data to this article can be found online at https://doi.org/10.1016/j.jhepr.2026.101997.

References

Author names in bold designate shared co-first authorship

[1] Hirode G, Choi HSJ, Chen CH, et al. Off-therapy response after nucleos(t)ide analogue withdrawal in patients with chronic hepatitis B: an international, multicenter, multiethnic cohort (RETRACT-B study). Gastroenterology 2022;162. 757–71.e754.
[2] Pol S, Nalpas B, Driss F, et al. Efficacy and limitations of a specific immunotherapy in chronic hepatitis B. J Hepatol 2001;34:917–921.
[3] Dembek C, Protzer U, Roggendorf M. Overcoming immune tolerance in chronic hepatitis B by therapeutic vaccination. Curr Opin Virol 2018;30: 58–67.
[4] Heim K, Neumann-Haefelin C, Thimme R, et al. Heterogeneity of HBV-specific $CD8^+$ T-cell failure: implications for immunotherapy. Front Immunol 2019;10:2240.
[5] **Ghany MG**, **Buti M**, **Lampertico P**, et al. Guidance on treatment endpoints and study design for clinical trials aiming to achieve cure in chronic hepatitis B and D: report from the 2022 AASLD-EASL HBV-HDV Treatment Endpoints Conference. Hepatology 2023;78:1654–1673.
[6] **Yan H**, **Zhong G**, Xu G, et al. Sodium taurocholate cotransporting polypeptide is a functional receptor for human hepatitis B and D virus. eLife 2012;1:e00049.
[7] Ni Y, Lempp FA, Mehrle S, et al. Hepatitis B and D viruses exploit sodium taurocholate co-transporting polypeptide for species-specific entry into hepatocytes. Gastroenterology 2014;146:1070–1083.
[8] Pontisso P, Ruvoletto MG, Gerlich WH, et al. Identification of an attachment site for human liver plasma membranes on hepatitis B virus particles. Virology 1989;173:522–530.
[9] **Glebe D**, **Urban S**, Knoop EV, et al. Mapping of the hepatitis B virus attachment site by use of infection-inhibiting preS1 lipopeptides and tupaia hepatocytes. Gastroenterology 2005;129:234–245.
[10] Barrera A, Guerra B, Notvall L, et al. Mapping of the hepatitis B virus pre-S1 domain involved in receptor recognition. J Virol 2005;79:9786–9798.
[11] Bian Y, Zhang Z, Sun Z, et al. Vaccines targeting preS1 domain overcome immune tolerance in hepatitis B virus carrier mice. Hepatology 2017;66:1067–1082.
[12] Pfefferkorn M, Bohm S, Schott T, et al. Quantification of large and middle proteins of hepatitis B virus surface antigen (HBsAg) as a novel tool for the identification of inactive HBV carriers. Gut 2018;67: 2045–2053.

[13] Alberti A, Cavalletto D, Chemello L, et al. Fine specificity of human antibody response to the PreS1 domain of hepatitis B virus. Hepatology 1990;12:199–203.
[14] **Wei J**, **Wang YQ**, **Lu ZM**, et al. Detection of anti-preS1 antibodies for recovery of hepatitis B patients by immunoassay. World J Gastroenterol 2002;8:276–281.
[15] Wang W, Zhou X, Bian Y, et al. Dual-targeting nanoparticle vaccine elicits a therapeutic antibody response against chronic hepatitis B. Nat Nanotechnol 2020;15:406–416.
[16] **Zakeri B**, **Fierer JO**, Celik E, et al. Peptide tag forming a rapid covalent bond to a protein, through engineering a bacterial adhesin. Proc Natl Acad Sci U S A 2012;109:E690–E697.
[17] Ko C, Chakraborty A, Chou WM, et al. Hepatitis B virus genome recycling and de novo secondary infection events maintain stable cccDNA levels. J Hepatol 2018;69:1231–1241.
[18] Li D, He W, Liu X, et al. A potent human neutralizing antibody Fc-dependently reduces established HBV infections. eLife 2017;6:e26738.
[19] Cargill T, Barnes E. Therapeutic vaccination for treatment of chronic hepatitis B. Clin Exp Immunol 2021;205:106–118.
[20] Thimme R, Wieland S, Steiger C, et al. CD8$^+$ T cells mediate viral clearance and disease pathogenesis during acute hepatitis B virus infection. J Virol 2003;77:68–76.
[21] Collier AY, Yu J, McMahan K, et al. Differential kinetics of immune responses elicited by Covid-19 vaccines. N Engl J Med 2021;385:2010–2012.
[22] McDonald I, Murray SM, Reynolds CJ, et al. Comparative systematic review and meta-analysis of reactogenicity, immunogenicity and efficacy of vaccines against SARS-CoV-2. NPJ Vaccin 2021;6:74.
[23] **Cargill T**, **Cicconi P**, Brown A, et al. HBV001: phase I study evaluating the safety and immunogenicity of the therapeutic vaccine ChAdOx1-HBV. JHEP Rep 2023;5:100885.
[24] Tak WY, Chuang WL, Chen CY, et al. Phase Ib/IIa randomized study of heterologous ChAdOx1-HBV/MVA-HBV therapeutic vaccination (VTP-300) as monotherapy and combined with low-dose nivolumab in virally-suppressed patients with CHB. J Hepatol 2024;81:949–959.
[25] **Chinnakannan SK**, **Cargill TN**, Donnison TA, et al. The design and development of a multi-HBV antigen encoded in chimpanzee adenoviral and modified vaccinia Ankara viral vectors; a novel therapeutic vaccine strategy against HBV. Vaccines (Basel) 2020;8:184.
[26] Su J, Brunner L, Ates Oz E, et al. Activation of CD4 T cells during prime immunization determines the success of a therapeutic hepatitis B vaccine in HBV-carrier mouse models. J Hepatol 2023;78:717–730.
[27] **Boni C**, **Fisicaro P**, Valdatta C, et al. Characterization of hepatitis B virus (HBV)-specific T-cell dysfunction in chronic HBV infection. J Virol 2007;81:4215–4225.
[28] Schuch A, Salimi Alizei E, Heim K, et al. Phenotypic and functional differences of HBV core-specific versus HBV polymerase-specific CD8$^+$ T cells in chronically HBV-infected patients with low viral load. Gut 2019;68:905–915.
[29] Keeble AH, Yadav VK, Ferla MP, et al. DogCatcher allows loop-friendly protein–protein ligation. Cell Chem Biol 2021;29:339. 50.e10.
[30] Dicks MDJ, Rose LM, Russell RA, et al. Modular capsid decoration boosts adenovirus vaccine-induced humoral immunity against SARS-CoV-2. Mol Ther 2022;30:3639–3657.
[31] Biswas S, Dicks MD, Long CA, et al. Transgene optimization, immunogenicity and *in vitro* efficacy of viral vectored vaccines expressing two alleles of *Plasmodium falciparum* AMA1. PLoS One 2011;6:e20977.
[32] Han WG, Unger WW, Wauben MH. Identification of the immunodominant CTL epitope of EGFP in C57BL/6 mice. Gene Ther 2008;15:700–701.
[33] Schirmbeck R, Bohm W, Fissolo N, et al. Different immunogenicity of H-2 Kb-restricted epitopes in natural variants of the hepatitis B surface antigen. Eur J Immunol 2003;33:2429–2438.
[34] Martin P, Dubois C, Jacquier E, et al. TG1050, an immunotherapeutic to treat chronic hepatitis B, induces robust T cells and exerts an antiviral effect in HBV-persistent mice. Gut 2015;64:1961–1971.
[35] Wettengel JM, Linden B, Esser K, et al. Rapid and robust continuous purification of high-titer hepatitis B virus for in vitro and in vivo applications. Viruses 2021;13:1503.
[36] Wing PA, Davenne T, Wettengel J, et al. A dual role for SAMHD1 in regulating HBV cccDNA and RT-dependent particle genesis. Life Sci Alliance 2019;2:e201900355.
[37] Battagliotti JM, Fontana D, Etcheverrigaray M, et al. Characterization of hepatitis B virus surface antigen particles expressed in stably transformed mammalian cell lines containing the large, middle and small surface protein. Antivir Res 2020;183:104936.
[38] Liu Z, Chen O, Wall JBJ, et al. Systematic comparison of 2A peptides for cloning multi-genes in a polycistronic vector. Sci Rep 2017;7:2193.
[39] **Urban S**, **Neumann-Haefelin C**, Lampertico P. Hepatitis D virus in 2021: virology, immunology and new treatment approaches for a difficult-to-treat disease. Gut 2021;70:1782–1794.
[40] Wedemeyer H, Aleman S, Brunetto M, et al. Bulevirtide monotherapy in patients with chronic HDV: efficacy and safety results through week 96 from a phase III randomized trial. J Hepatol 2024;81:621–629.
[41] Allweiss L, Volmari A, Suri V, et al. Blocking viral entry with bulevirtide reduces the number of HDV-infected hepatocytes in human liver biopsies. J Hepatol 2024;80:882–891.
[42] Mohsen MO, Zha L, Cabral-Miranda G, et al. Major findings and recent advances in virus-like particle (VLP)-based vaccines. Semin Immunol 2017;34:123–132.
[43] Fernandes P, Wang Y, Doisne JM, et al. Functional immune responses induced by a capsid assembly modulator in chronic hepatitis B virus-infected humanized mice. Cell Host Microbe 2026;34:116. 30.e117.
[44] **Feld JJ**, **Lok AS**, Zoulim F. New perspectives on development of curative strategies for chronic hepatitis B. Clin Gastroenterol Hepatol 2023;21:2040–2050.
[45] Ji Y, Le Bert N, Lai-Hung Wong G, et al. The impact of hepatitis B surface antigen reduction via small interfering RNA treatment on natural and vaccine (BRII-179)-induced hepatitis B virus-specific humoral and cellular immune responses. Gastroenterology 2025;169:136–149.
[46] Agarwal K, Yuen M-F, Roberts S, et al. OS-031 Imdusiran (AB-729) administered every 8 weeks for 24 weeks followed by the immunotherapeutic VTP-300 maintains lower HBV surface antigen levels in NA-suppressed CHB subjects than 24 weeks of imdusiran alone. J Hepatol 2024;80:S26–S27.
[47] Asselah T, Chattergoon MA, Jucov A, et al. A phase 2 trial of tobevibart plus elebsiran in hepatitis D. N Engl J Med 2026;394:343–353.
[48] Salimzadeh L, Shah S, La D, et al. SAT-536 uptake and T cell stimulation by anti-HBs monoclonal antibody BJT-778-rHBsAg immune complexes. J Hepatol 2024;80:S352.
[49] Asselah T, Chulanov V, Lampertico P, et al. Bulevirtide combined with pegylated interferon for chronic hepatitis D. N Engl J Med 2024;391:133–143.
[50] Wedemeyer H, Aleman S, Blank A, et al. LBO-004 Final results of MYR301: a randomised phase 3 study evaluating the efficacy and safety of up to 144 weeks of bulevirtide monotherapy for chronic hepatitis delta and 96 weeks of posttreatment follow-up. J Hepatol 2025;82:S10–S11.

Keywords: Therapeutic vaccine; HBV; HDV; Protein superglue; Antibodies; T cells.
Received 11 March 2026; received in revised form 5 August 2026; accepted 10 August 2026; Available online 17 August 2026

Journal of Hepatology, Volume *8*

Supplemental information

Therapeutic vaccination strategy targeting the PreS1 region of L-HBsAg for HBV/HDV co-infection

Rebecca A. Russell, James Lok, James M. Harris, Senko Tsukuda, Louisa M. Rose, Bethany G. Charlton, Ivana Carey, Kosh Agarwal, Peter A.C. Wing, Sumi Biswas, Jane A. McKeating, and Matthew D.J. Dicks

Therapeutic vaccination strategy targeting the PreS1 region of L-HBsAg for HBV/HDV co-infection

Rebecca A. Russell, James Lok, James M. Harris, Senko Tsukuda, Louisa M. Rose, Bethany G. Charlton, Ivana Carey, Kosh Agarwal, Peter A.C. Wing, Sumi Biswas, Jane A. McKeating, Matthew D. J. Dicks

Table of contents

Supplementary methods……………………………………………………………………2

Supplementary figures…………………………………………………………..………..4

Supplementary methods

Recombinant adenovirus HBV transgene expression

Transgene expression from HBV-encoding vectors was determined by infecting 293A cells, seeded at 1.25E+6 in 6-well plates, with the Ad vectors at a multiplicity of infection of 100. The next day cells were harvested and 5E+5 cells were lysed in reducing 4x Laemmli sample buffer (Bio-Rad, UK) with 300mM 1,4-dithiothreitol (Merck, UK) at 95ºC for 10 minutes. Samples were separated on a 4-12% Bis-Tris gel (Life Technologies, UK) and transferred to a 0.2 μm nitrocellulose membrane (Bio-Rad, UK). Western blots were carried out using the iBind system (Life Technologies, UK). The following antibodies were used: anti-β-Actin rabbit monoclonal antibody (mAb) clone D6A8 at 1:500 (Cell Signaling Technologies, The Netherlands; anti-Ad5 hexon mouse mAb clone 65H6 at 1:500 (Life Technologies, UK); anti-S-HBsAg mouse mAb clone 1837 at 1:200 (Native Antigen, UK); anti-PreS1 mouse mAb clone AP1 at 1:200 (Santa Cruz Biotechnology, USA); anti-PreS2 mouse mAb clone S26 at 1:200 (Santa Cruz Biotechnology, USA); anti-Core mouse mAb clone 10E11 at 1:200 (Santa Cruz Biotechnology, USA). Goat anti-mouse IgG conjugated to alkaline phosphatase (STAR117A at 1:1000, Bio-Rad, UK) was used as a secondary for all mouse primary antibodies and goat anti-rabbit conjugated to alkaline phosphatase (A3687 at 1:1000, Merck, UK) was used as a secondary for the anti-β-Actin rabbit primary antibody. The signal was developed using BCIP/NBT solution (Merck, UK).

Flow cytometry protocol and antibody cocktails:

Cells were stained with Fixable Live/Dead Aqua (1:1000 Life Technologies, UK) and Fc receptors were blocked with 2.5 µg/mL CD16/CD32 Fc block (BD Biosciences, UK) before being stained with an extracellular antibody cocktail diluted in FACS wash buffer (PBS with 2 mM EDTA and 0.5% bovine serum albumin) (anti-mouse CD3 APC-Fire750, clone 17A2, 1:40, BioLegend, USA; anti-mouse CD4 PE-Dazzle594, clone GK1.5, 1:200, BioLegend, USA; anti-mouse CD8a BB700, clone 53-6.7, 1:80, BD Biosciences, UK; anti-mouse CD14 BV510, clone Sa14-2, 1:80, BioLegend, USA and anti-mouse CD19 BV510, clone 1D3, 1:200, BioLegend, USA). Cells were fixed in Cytofix/Cytoperm (BD Biosciences, UK) before being stained with an intracellular antibody cocktail diluted in Perm/Wash buffer (BD Biosciences, UK) (anti-mouse IFNγ APC, clone XMG1.2, 1:160, BioLegend, USA; anti-mouse IL-2 PE, clone JES6-5H4, 1:80, BioLegend, USA and anti-mouse TNFα FITC, clone MP6-XT22, 1:400, BioLegend, USA).

Preparation of HepG2-NTCP cells for the HBV neutralization assay

For the HBV neutralization assay HepG2-NTCP cells were seeded at 1E+4/well in DMEM (Life Technologies, UK) with 10% Foetal Calf Serum (Gibco, UK), 1% Non-Essential Amino Acids (Life Technologies, UK) and 1% Penicillin & Streptomycin (Life Technologies, UK) (complete medium) in collagen-coated (Sigma) flat-bottomed 96-well plates (Corning, USA). The next day the medium was replaced with complete medium supplemented with 2.5% DMSO (Merck, UK) and the cells incubated for 72 hours. The medium from the HepG2-NTCP cells was removed and replaced with 100 μL complete medium supplemented with 2.5% DMSO and 4% Polyethylene glycol (PEG8000) (Merck, UK). 10 μL of the reporter virus/serum dilution series was added to the cells and the infection allowed to proceed for 16 hours. The viral inocula was removed, cells washed twice with 100 μL PBS, and the medium replaced with 100μL complete medium supplemented with 2.5% DMSO. After 3 days the medium was

replenished. After a further 3 days the Gaussia luciferase signal was detected to determine the level of infection.

Preparation of HDV

Twenty-four hours prior to transfection, Huh-7 cells were seeded in complete medium at a density of 6E+6 cells/T75 flask. The next day, cells were transfected with 5 μg of pSVLD3 and 5 μg of pT7HB2.7 plasmids using 30 μL of FuGENE-HD reagent (Promega, UK). Following overnight incubation, cells were washed twice with PBS and the medium was replaced with 12.5 mL of complete medium. Supernatant was collected at 3-, 6-, 9-, and 12-days post-transfection, then concentrated to $1/50^{th}$ of the original supernatant volume using Ultra Centrifugal Filter 50 kDa (Merck, UK) and used as HDV inoculum. To quantify genome copy number, RNA from 3 μL of concentrated HDV inoculum was extracted using a Viral RNA Mini Kit (QIAGEN, Germany), and reverse transcribed using a cDNA synthesis kit (PCR Biosystems, UK), according to the manufacturer's protocol (25°C, 10 min; 42°C, 15 min; 48°C, 15 min; 85°C, 10 min). Gene expression was quantified using a SyGreen Blue Mix (PCR Biosystems, UK) using a qPCR program of 95°C, 2 min; 45 cycles of 95°C, 5 sec; 60°C, 30 sec, using primers specific for HDV <5'-TCTTCCTCGGTCAACCTCTT-3' and 5'-ACAAGGAGAGGCAGGATCAC-3'>. Genome copy number was enumerated from a standard curve of pSVLD3 plasmid of known concentration.

Preparation of HepG2-NTCP cells for the HDV neutralisation assay

HepG2-NTCP cells were seeded at a density of 1.25E+4 cells/well on collagen coated 96 well plates and cultured in complete medium. Next day the medium was replaced with complete medium containing 2.5% DMSO for a further 72h. Test sera were pre-inoculated with HDV (MOI of 50 genome copies per cell) in the presence of 4% PEG8000 and 2.5% DMSO at 37°C for 1h, then added to the cells for 24h infection. Viral inoculum was removed, and cells washed three times with PBS. Infected cells were maintained with complete medium containing 2.5% DMSO in 5% CO_2 and 18% O_2, changing media every 3 days. Six days post infection, cells were fixed with 4% PFA and the number of HDV-infected cells determined by immunofluorescence.

The number of HDV-infected cells was quantified by manual counting of GFP positive cells in 6 images from 3 independent infections (2 images/infection), and significance assessed using Kruskal Wallis with Dunn's test for multiple comparisons, comparing all test conditions to the HDV-infected control, *$p < 0.05$, ***$p < 0.001$, ns = non-significant.

Supplementary figures

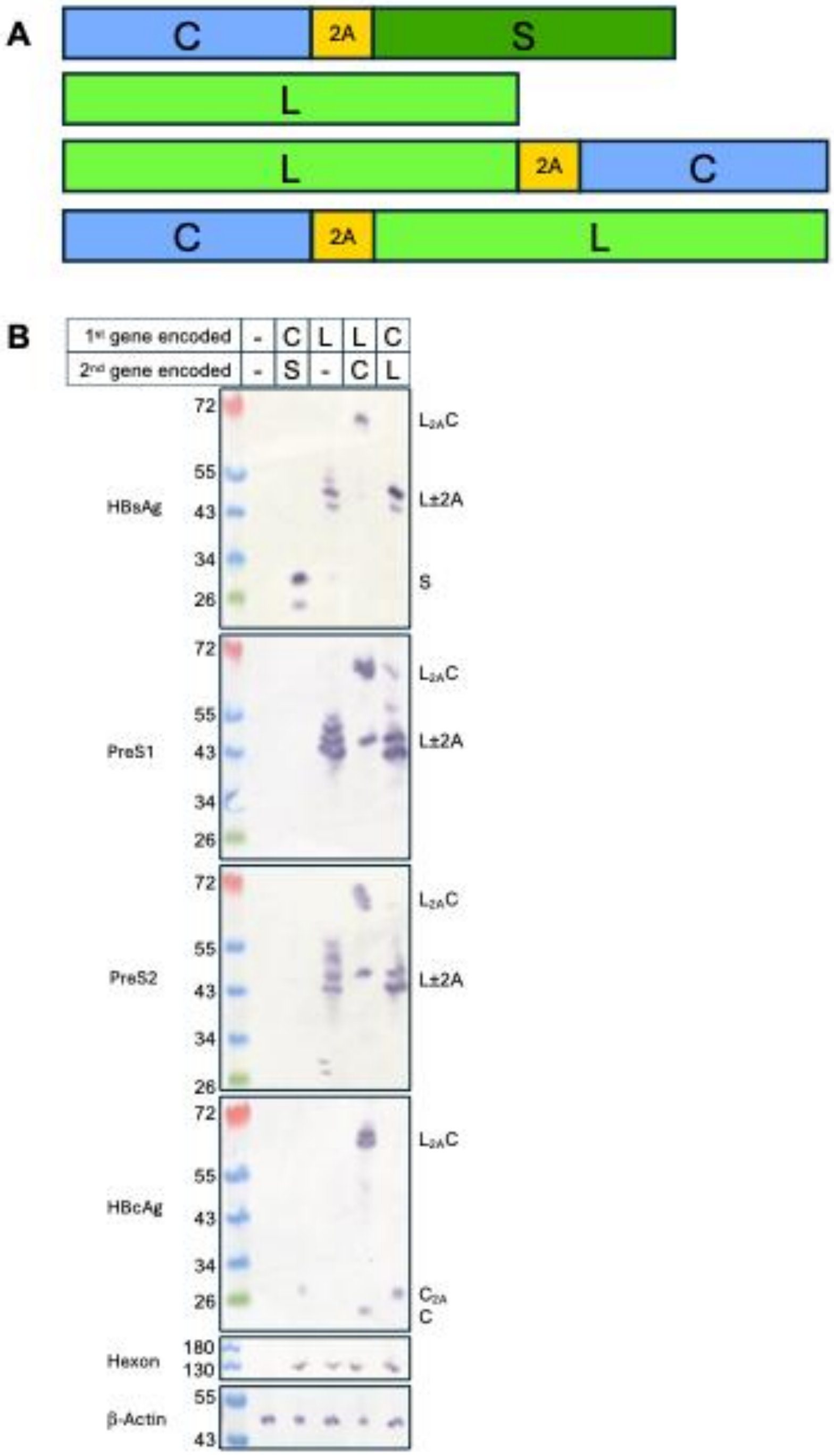

Fig. S1. Encoded HBV genes. (A) Schematic of the different HBV genes encoded in the Ad vectors. Vectors were designed to express HBcAg (C), the S-HBsAg (S) or the L-HBsAg (L), separated, where necessary, by a GSG linker and an F2A sequence. For optimal expression and immunogenicity a Kozak sequence followed by a truncated shark Invariant chain (SIi) and a GSGGSG linker were inserted up stream of the first open reading frame. (B) HEK293A cells were infected with Ad vectors at a multiplicity of infection of 100. 24 h post-transfection, cells were lysed and the lysates were analysed by Western blot using mouse anti-HBsAg, mouse anti-PreS1, mouse anti-PreS2 and mouse anti-HBcAg antibodies. Blots probed with mouse anti-Ad5 hexon and rabbit anti-beta-actin served as loading controls. Lane 1: cell lysate from un-transduced cells, lanes

2 to 5: cell lysates from cells transduced with Ad encoding HBV genes as detailed in the grid above the first blot.

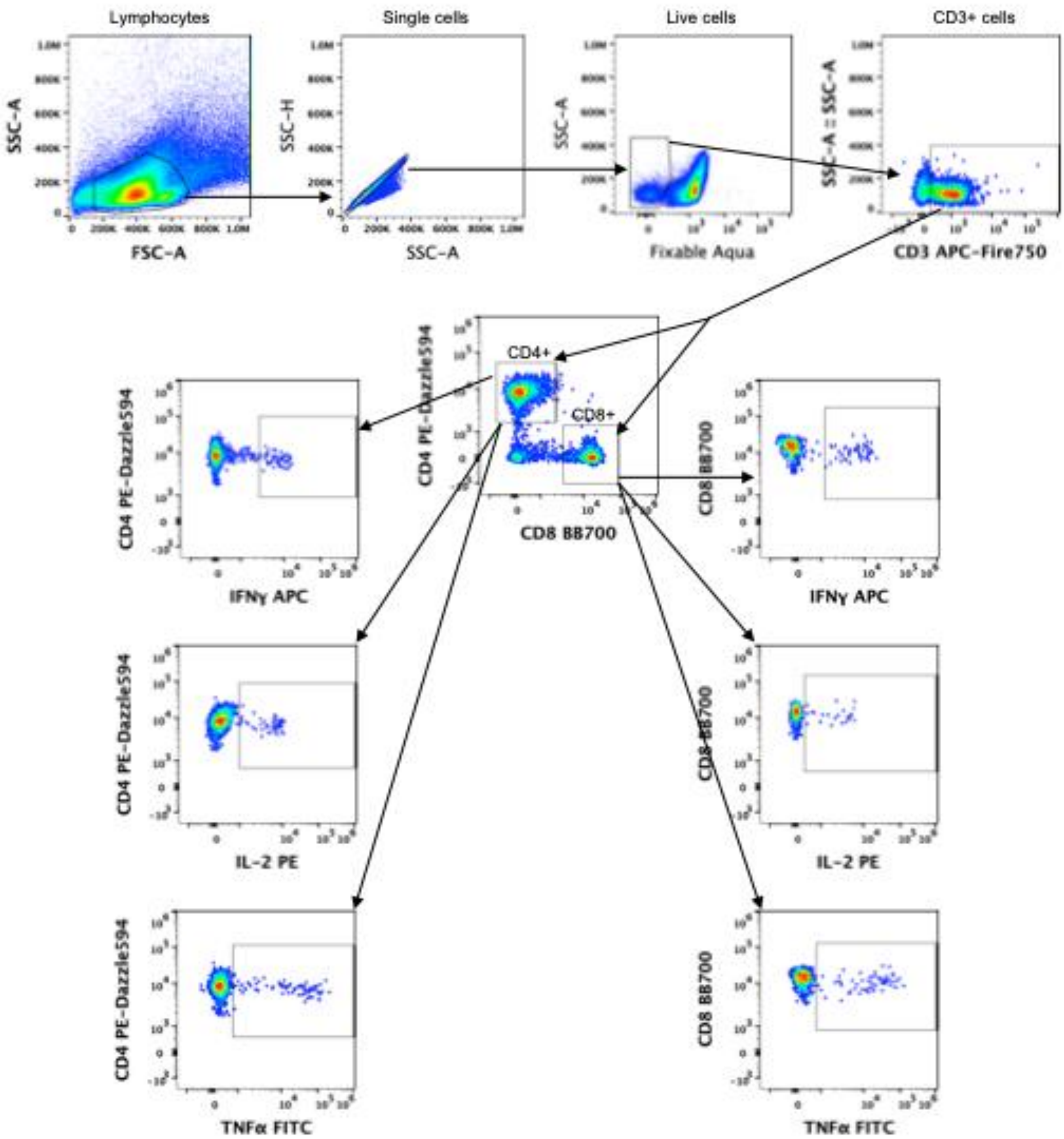

Fig. S2. Flow cytometry gating strategy. Flow cytometry was performed on an Attune-NxT and data was analyzed with FlowJo version 10.10. The gating strategy for all samples is shown. Lymphocytes were gated on forward and side scatter area (FSC-A and SSC-A, respectively). Single lymphocytes were then gated on SSC area (SSC-A) and height (SSC-H). Dead cells, CD14+ monocytes and CD19+ B cells were then removed by fixable aqua, CD14 and CD19 staining. CD3+ T-cells were then gated on CD3 APC-Fire and SSC-A. CD4+ and CD8+ cells were separated on CD4 PE-Dazzle594 and CD8 BB700 staining. Within the CD4+ and CD8+ populations IFNgamma, IL-2 and TNFalpha expression was assessed with IFNgamma APC, IL-2 PE and TNFalpha FITC gating, respectively.

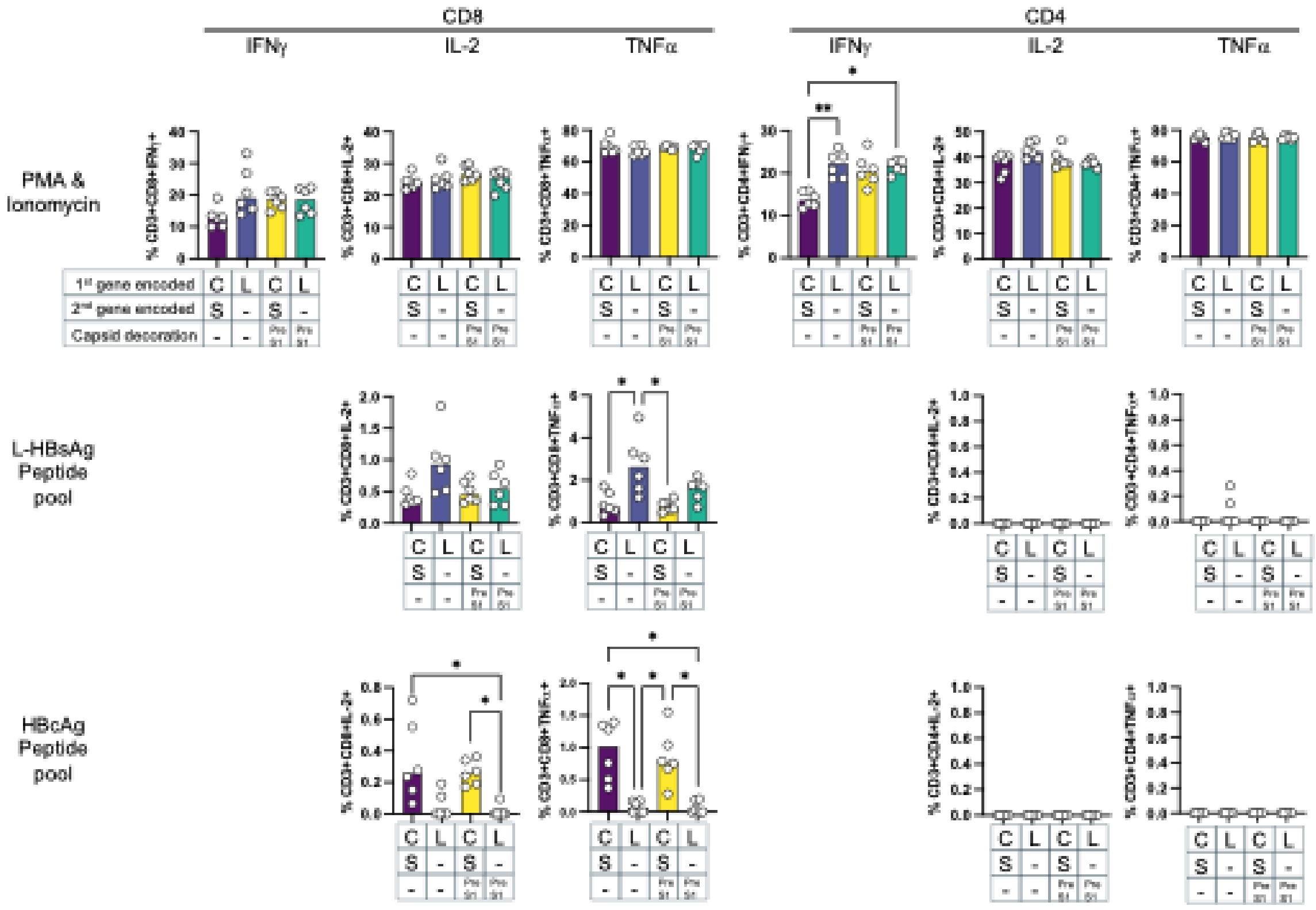

Fig. S3. Cytokine responses from CD4+ and CD8+ T-cells from the experiment shown in Fig. 4. Percentages of cytokine producing (IFNgamma, IL-2 and TNFalpha) CD3+ CD4+ and CD3+ CD8+ T-cells at D35 after stimulation with either phorbol 12-myristate 13-acetate (PMA) and Ionomycin or peptide pools of L-HBsAg or HBcAg. Median responses are shown. Statistical analyses performed by Kruskal-Wallis with Dunn's test for multiple comparisons, $^{**}p<0.01$, $^{*}p<0.05$, non-significant comparisons are not shown. For HBcAg peptide pools, comparisons were only carried out between groups encoding the relevant antigens.

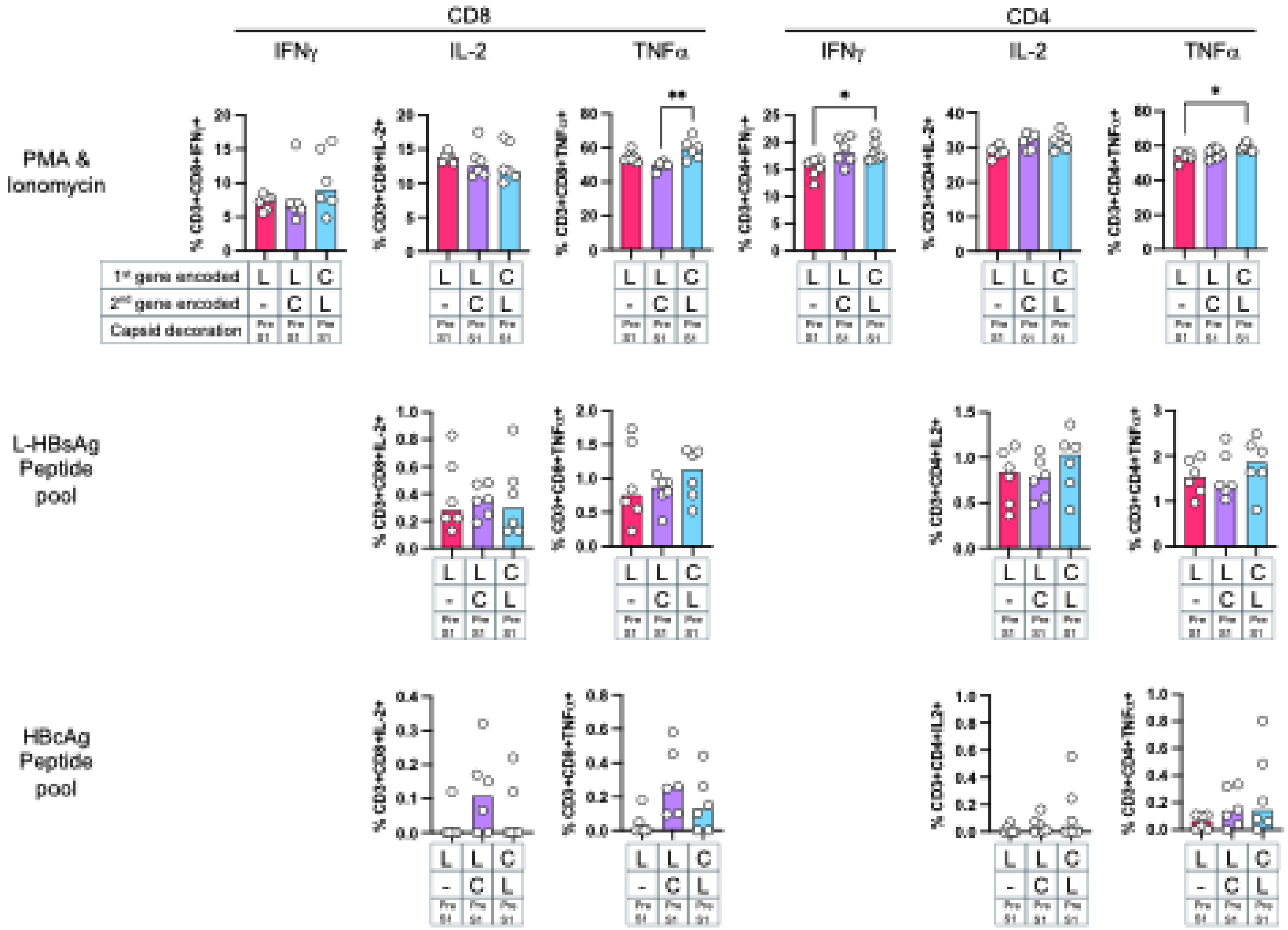

Fig. S4. Cytokine responses from CD4+ and CD8+ T-cells from the experiment shown in Fig. 5. Percentages of cytokine producing (IFNgamma, IL-2 and TNFalpha) CD3+ CD4+ and CD3+ CD8+ T-cells at D35 after stimulation with either phorbol 12-myristate 13-acetate (PMA) and Ionomycin or peptide pools of L-HBsAg or HBcAg. Median responses are shown. Statistical analyses performed by Kruskal-Wallis with Dunn's test for multiple comparisons, $^{**}p<0.01$, $^{*}p<0.05$, non-significant comparisons are not shown. For HBcAg peptide pools, comparisons were only carried out between groups encoding HBcAg.
