# Supplementary material for: Therapeutic vaccination strategy targeting the PreS1 region of L-HBsAg for HBV/HDV co-infection
Source: JHEP Rep. 2026 Aug 17;8(11):101997. doi: 10.1016/j.jhepr.2026.101997 (PMC13659496; doi:10.1016/j.jhepr.2026.101997)
Supplement: Supplementary file 4 [file mmc2.docx]

**JHEP Reports**

**CTAT methods**

Tables for a “Complete, Transparent, Accurate and Timely account” (CTAT) are now mandatory for all revised submissions. The aim is to enhance the reproducibility of methods.

- Only include the parts relevant to your study
- Refer to the CTAT in the main text as ‘Supplementary CTAT Table’
- Do not add subheadings
- Add as many rows as needed to include all information
- Only include one item per row

**If the CTAT form is not relevant to your study, please outline the reasons why:**

1.1 **Antibodies**

| **Name** | **Citation** | **Supplier** | **Cat no.** | **Clone no.** |
| --- | --- | --- | --- | --- |
| APC/Fire™ 750 anti-mouse CD3 | **N/A Commercial** | Biolegend | 100248 | 17A2 |
| PE/Dazzle™ 594 anti-mouse CD4 | **N/A Commercial** | Biolegend | 100456 | GK1.5 |
| BB700 Rat Anti-Mouse CD8a | **N/A Commercial** | BD Bioscienes | 566409 | 53-6.7 |
| Brilliant Violet 510™ anti-mouse CD14 | **N/A Commercial** | Biolegend | 123323 | Sa14-2 |
| Brilliant Violet 510™ anti-mouse CD19 | **N/A Commercial** | Biolegend | 152423 | ID3 |
| APC anti-mouse IFN-γ | **N/A Commercial** | Biolegend | 505810 | XMG1.2 |
| FITC anti-mouse TNF-α | **N/A Commercial** | Biolegend | 506304 | MP6-XT22 |
| PE anti-mouse IL-2 | **N/A Commercial** | Biolegend | 503808 | JES6-5H4 |
| Mouse anti-HBsAg | **N/A Commercial** | The NativeAntigen Company | MAB12231-100 | 1837 |
| Mouse anti-PreS1 | **N/A Commercial** | Santa Cruz Biotechnology | sc-57761 | AP1 |
| Mouse anti-PreS2 | **N/A Commercial** | Santa Cruz Biotechnology | sc-23944 | S 26 |
| Mouse anti-HBcAg | **N/A Commercial** | Santa Cruz Biotechnology | sc-23947 | 1.00E+12 |
| Mouse anti-Hexon | **N/A Commercial** | ThermoFisher Scientific | LF-MA0177 | 65H6 |
| Rabbit anti-β-Actin | **N/A Commercial** | Cell Signalling Technology | #8457 | D6A8 |
| Goat anti-mouse IgG | **N/A Commercial** | BioRad | STAR117A | Polyclonal |
| Goat anti-rabbit IgG | **N/A Commercial** | Merck | A3687 | Polyclonal |
| Mouse anti-HDAg | **N/A Commercial** | Kerfast | EHD002 | FD5C4 |
| Alexa Fluor™ 488 goat anti-mouse igG | **N/A Commercial** | ThermoFisher Scientific | A-11001 | Polyclonal |
| Rat-anti mouse IFN-γ | **N/A Commercial** | Mabtech | 3321-3-1000 | AN18 |
| Biotinylated rat-anti mouse IFN-γ | **N/A Commercial** | Mabtech | 3321-6-250 | R4-6A2 |

1.2 **Cell lines**

| **Name** | **Citation** | **Supplier** | **Cat no.** | **Passage no.** | **Authentication test method** |
| --- | --- | --- | --- | --- | --- |
| **293A** | **N/A Commercial** | **ThermoFisher Scientific** | **R70507** | **Less than 20** |  |
| **Trex-293** | **N/A Commercial** | **ThermoFisher Scientific** | **R71007** | **Less than 20** |  |
| **EXPI-CHO-S** | **N/A Commercial** | **ThermoFisher Scientific** | **A29127** | **Less than 20** |  |
| **HepG2-NTCP** | Ni Y, Lempp FA, Mehrle S, Nkongolo S, Kaufman C, Falth M, Stindt J, et al. Hepatitis B and D viruses exploit sodium taurocholate co-transporting polypeptide for species-specific entry into hepatocytes. Gastroenterology 2014;146:1070-1083. |  |  |  |  |

1.3 **Organisms**

| **Name** | **Citation** | **Supplier** | **Strain** | **Sex** | **Age** | **Overall n number** |
| --- | --- | --- | --- | --- | --- | --- |
| **Mouse** | **N/A Commercial** | **Envigo** | **C57BL/6JOlaHsd 5706F** | **Female** | **Purchased at 6-8 weeks, immunised 8 days after arrival** | **N = 6 mice /vaccination group** |

1.4 **Sequence based reagents**

| **Name** | **Sequence** | **Supplier** |
| --- | --- | --- |
| **HepB PreS1 sequence** | genotype D, serotype ayw NC_003977, codon optimised for mammalian expression. QNLSTSNPLGFFPDHQLDPAFRANTANPDWDFNPNKDTWPDANKVGAGAFGLGFTPPHGGLLGWSPQAQGILQTLPANPPPASTNRQSGRQPTPLSPPLRNTHPQA | **Thermofisher Scientific, geneart** |

1.5 **Biological samples**

| **Description** | **Source** | **Identifier** |
| --- | --- | --- |
| **Serum from HBV positive individuals** | King's College Hospital Liver Research Biobank | **Anonymised patient number** |
| **Spleen, serum** | Mice listed in 1.3 post vaccination | **In house ear tag numbering system** |

1.6 **Deposited data**

| **Name of repository** | **Identifier** | **Link** |
| --- | --- | --- |
| **N/A** |  |  |

1.7 **Software**

| **Software name** | **Manufacturer** | **Version** |
| --- | --- | --- |
| **GraphPad Prism** | **GraphPad Software LLC** | **10.0.0** |
| **FlowJo** | **FloJo LLC** | **10.10.0** |
| **AID Classic** | **AID Diagnostica** | **7** |
|  |  |  |

1.8 **Other (*e.g*. drugs, proteins, vectors etc.)**

| **Description** | **Citation** | **Source** |
| --- | --- | --- |
| Ad-DogTag | Dicks MDJ, Rose LM, Russell RA, Bowman LAH, Graham C, Jimenez-Guardeno JM, Doores KJ, et al. Modular capsid decoration boosts adenovirus vaccine-induced humoral immunity against SARS-CoV-2. Mol Ther 2022;30:3639-3657. | **Created in house using standard techniques** |
| rHBV-gLuc | Wing PA, Davenne T, Wettengel J, Lai AG, Zhuang X, Chakraborty A, D'Arienzo V, et al. A dual role for SAMHD1 in regulating HBV cccDNA and RT-dependent particle genesis. Life Sci Alliance 2019;2 | **Created in house using standard techniques** |
| MyrcludexB (Bulevirtide™, Hepcludex™), |  | **Professor Stephan Urban**  **Heidelberg University** |

1.9 **Please provide the details of the corresponding methods author for the manuscript:**

**Dr Rebecca A. Russell**

**Address: SpyBiotech Limited, 7600 The Quorum, Alec Issigonis Way, Oxford Business Park North, Oxford, OX4 2JZ**

**Phone: +44 (0)1865 582088**

**Email: rebecca.moore@spybiotech.com**

**2.0	Please confirm for randomised controlled trials all versions of the clinical protocol are included in the submission. These will be published online as supplementary information.**

**N/A**
